# Multimaterial Embedded 3D Printing of Composite Reinforced Soft Actuators

**DOI:** 10.34133/research.0122

**Published:** 2023-04-18

**Authors:** Zhenhua Wang, Boyu Zhang, Qu He, Hao Chen, Jizhe Wang, Yuan Yao, Nanjia Zhou, Weicheng Cui

**Affiliations:** ^1^ Key Laboratory of Coastal Environment and Resources of Zhejiang Province, School of Engineering, Westlake University, Hangzhou, Zhejiang Province, China.; ^2^ Institute of Advanced Technology, Westlake Institute for Advanced Study, Hangzhou, Zhejiang Province, China.; ^3^ Research Center for Industries of the Future, and Key Laboratory of 3D Micro/Nano Fabrication and Characterization of Zhejiang Province, School of Engineering, Westlake University, Hangzhou, Zhejiang Province, China.

## Abstract

Soft pneumatic actuators (SPAs) have attracted enormous attention in the growing field of robotics. Among different SPAs, composite reinforced actuators (CRAs) are widely used because of their simple structure and high controllability. However, multistep molding, a time-consuming method, is still the predominant fabrication method. Here, we propose a multimaterial embedded printing method (ME3P) to fabricate CRAs. In comparison with other 3-dimensional printing methods, our method improves fabrication flexibility greatly. Via the design and fabrication of the reinforced composites’ patterns and different geometries of the soft body, we demonstrate actuators with programmable responses (elongation, contraction, twisting, bending, and helical and omnidirectional bending). Finite element analysis is employed for the prediction of pneumatic responses and the inverse design of actuators based on specific actuation needs. Lastly, we use tube-crawling robots as a model system to demonstrate our ability to fabricate complex soft robots for practical applications. This work demonstrates the versatility of ME3P for the future manufacturing of CRA-based soft robots.

## Introduction

Inspired by nature, soft actuators have attracted great attention for applications in delicate object manipulation, safe human–robot interactions, and medical robots for diagnosis and surgery thanks to their inherent mechanical compliance [1–5]. Among different types of soft actuators, soft pneumatic actuators (SPAs) present strong advantages in high workload, excellent reversibility and controllability, as well as fast actuation speed [6,7]. Soft composite reinforced actuators (CRAs), as a branch of SPAs, employ a combination of inextensible materials with soft elastomer materials, greatly expanding the actuator functionalities while still adopting simple geometries. Conventional CRAs are fabricated by wrapping the soft inflating materials (e.g., silicone rubber) with stiff fibers (Kevlar fiber [6–9], thermoplastic polyurethanes [10], resin [11,12], and others). Compared with other types of SPAs (especially PneuNet actuators [13]), CRAs feature simple structural design, uniform stress distribution, and convenient modeling and programming methods [14]. The fiber diameters, winding pitches, and orientations are readily programmable to design and manufacture application-specific actuators that enable complex motions [14].

Regarding the manufacturing of CRAs, multistep molding is the most used strategy [11,15,16]. However, it suffers from limited design freedom while requiring much manual intervention, which poses challenges in accurate assembly [10]. In addition, the winding of fibers on the soft inflating surface typically are limited to only simple patterns (fibers with different winding angle). Such methods are insufficient for CRAs with complex shape morphing ability, which are substantial for functional soft actuators [17,18]. Three-dimensional (3D) printing, as an emerging digital manufacturing approach, has been demonstrated for the rapid fabrication of CRAs with complex reinforced patterns. Most previous reports of 3D printed CRAs predominantly focus on the manual assembly of fiber layers with molded silicone elastomer bodies [11,19] printed typically via vat photopolymerization. These methods still involve inaccurate and time-consuming manual casting work. Also, the materials employed in vat photopolymerization are limited to light-curing materials (resins, hydrogels, and others) [20]. Direct ink writing (DIW), on the other hand, could overcome these problems to become a rapid and digital fabrication method for CRAs with wider material choices [10,21 ,22]. For example, Schaffner et al. [21] use DIW to print stiff fiber patterns with different orientation angles onto a layer of silicone on a rotating cylindrical shaft. Although fibers with various patterns can be fabricated, the overall geometries of the inflating bodies are highly restricted by the shape of the shaft. Embedded 3D printing, featuring printing the structural and functional inks in a common supporting matrix, could overcome such shape limitations [23,24], and it has been used to fabricate PneuNet actuators generating single motions [25–27] and fully functional robots based on them. However, implemented in the normal Cartesian machine coordinate system, embedded 3D printing faces challenges in the freeform patterning of fibers onto arbitrary 3D geometries. The fabrication of more complex pneumatics CRAs, e.g., the shape-morphing CRAs, demands the development of embedded 3D printable materials featuring different mechanical properties and improved multiaxis printing platform.

In this work, for the first time, we propose a novel multimaterial embedded 3D printing (ME3P) method for CRAs, which enables the monolithic design and manufacturing of arbitrary-shaped complex actuator bodies with inner channels as well as the facile patterning of complex reinforced materials on actuator surfaces. The mechanical and rheological properties of the hard, soft, and matrix materials are investigated to optimize the design and manufacturing of the CRAs. The process parameters of the ME3P are investigated to help understand the filament morphology to optimize the print quality and resolution. We demonstrate actuators with different reinforced composite patterns to generate programmable pneumatic responses. We also demonstrate the CRAs with shape morphing ability and local compliant mechanisms. A finite element analysis (FEA) data-driven method is utilized for the inverse design of actuators. Finally, 2 crawling robots capable of moving through forked tubes both in air and in water are printed as demonstrations.

## Results and Discussion

### Printing method

**Fig. 1.**
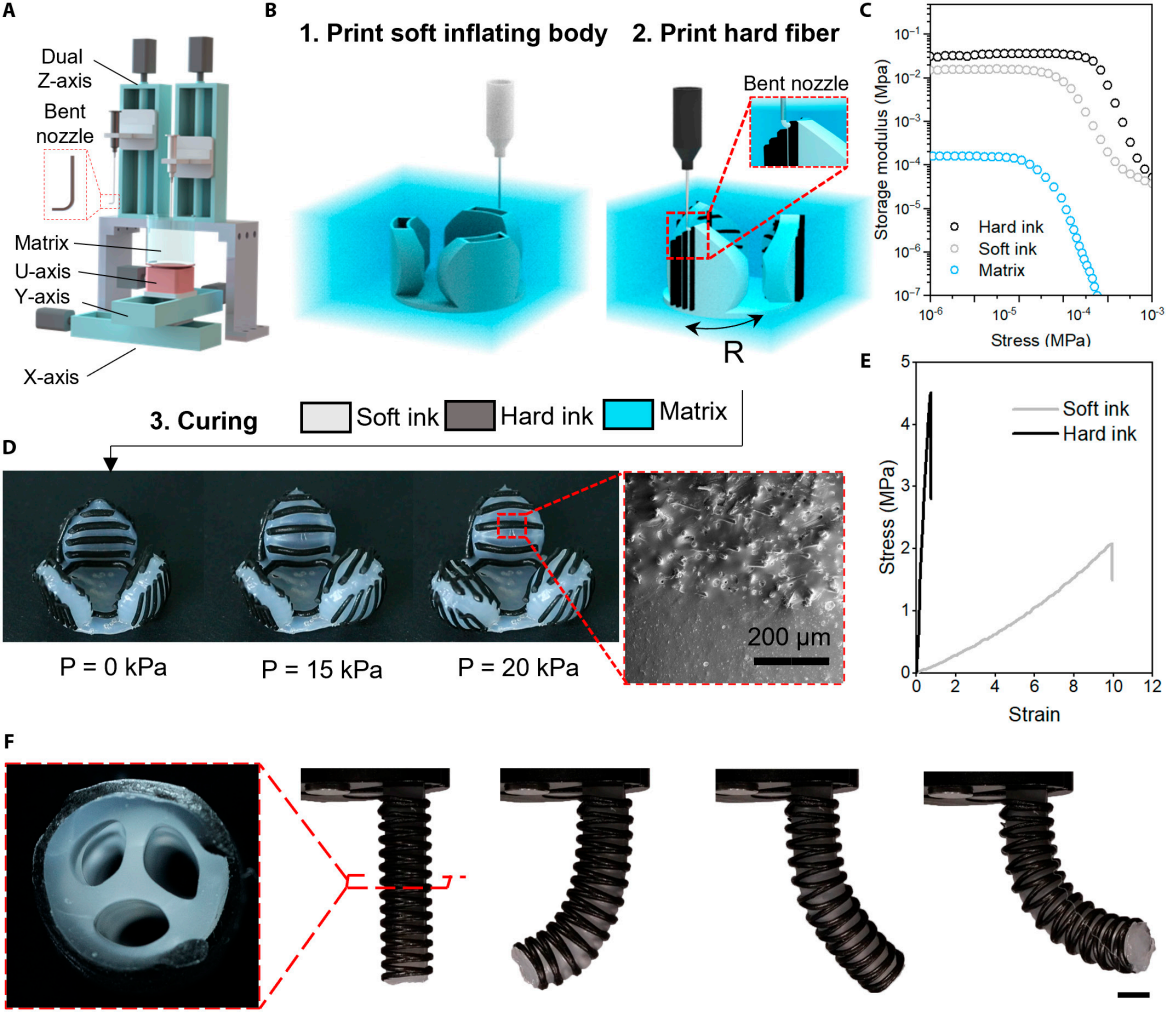
Overview of multimaterial embedded printing for soft composite actuators and robots. (A) Dual-material multiaxis embedded 3D printing setup. (B) Schematic diagram illustrating the fabrication processes of a soft pneumatic flower. (C) The storage moduli of hard and soft inks for embedded printing are ~2 orders of magnitude higher than the matrix. (D) The printed flower blooms at the inflating pressure of ~20 kPa (left, scale bar: 10 mm) and an SEM image showing the interfaces between the pure PDMS material and PDMS-CF composite. (E) Strain–stress curve of soft and hard inks showing the dramatical difference between the elastic moduli of them. (F) An omnidirectional bending actuator with 3 embedded channels (left) can bend in different directions under different inflating states of 3 channels (right).

The structure of our printing system is shown in Fig. 1A. For our first demonstration, we printed a pneumatic flower via a 2-step process (Fig. 1B and Movie S1): First, the soft ink was used to print flower petals with air channels (Fig. S1) in the matrix; second, the hard ink was conformally printed on both sides of the petals to control the shape morphing upon inflation. A rotary axis (U-axis) was added to print stiff reinforced patterns on the petal surfaces. The nozzle tip in step 2 was bent to 90° to ensure that it is perpendicular to the petal surfaces at the printing position (Fig. 1A and B). As reported in previous works [28,29], for successful embedded printing, the storage moduli of inks should be 1 or 2 orders of magnitude higher than those of the matrix materials (Fig. 1C). For this study, we selected carbomer gel as the matrix material, whose rheological properties have been studied in our previous work [27]. The blooming of the cured flower agrees with the designed objective, showing that the printed hard patterns present good reinforced performances (Fig. 1D and Movie S2). We next demonstrate an omnidirectional bending actuator featuring a soft cylindrical inflating body with 3 independent inner channels (Fig. 1F, left). Subsequently, a stiff reinforced woven at the cross angle of ±20° with respect to the axial orientation of the cylinder was printed to limit its radial inflation. The pressures of 3 channels were controlled by 3 independent air supplies. Figure 1F demonstrates its ability of omnidirectional bending motions.

### Rheological and mechanical properties of the ink and matrix

**Fig. 2.**
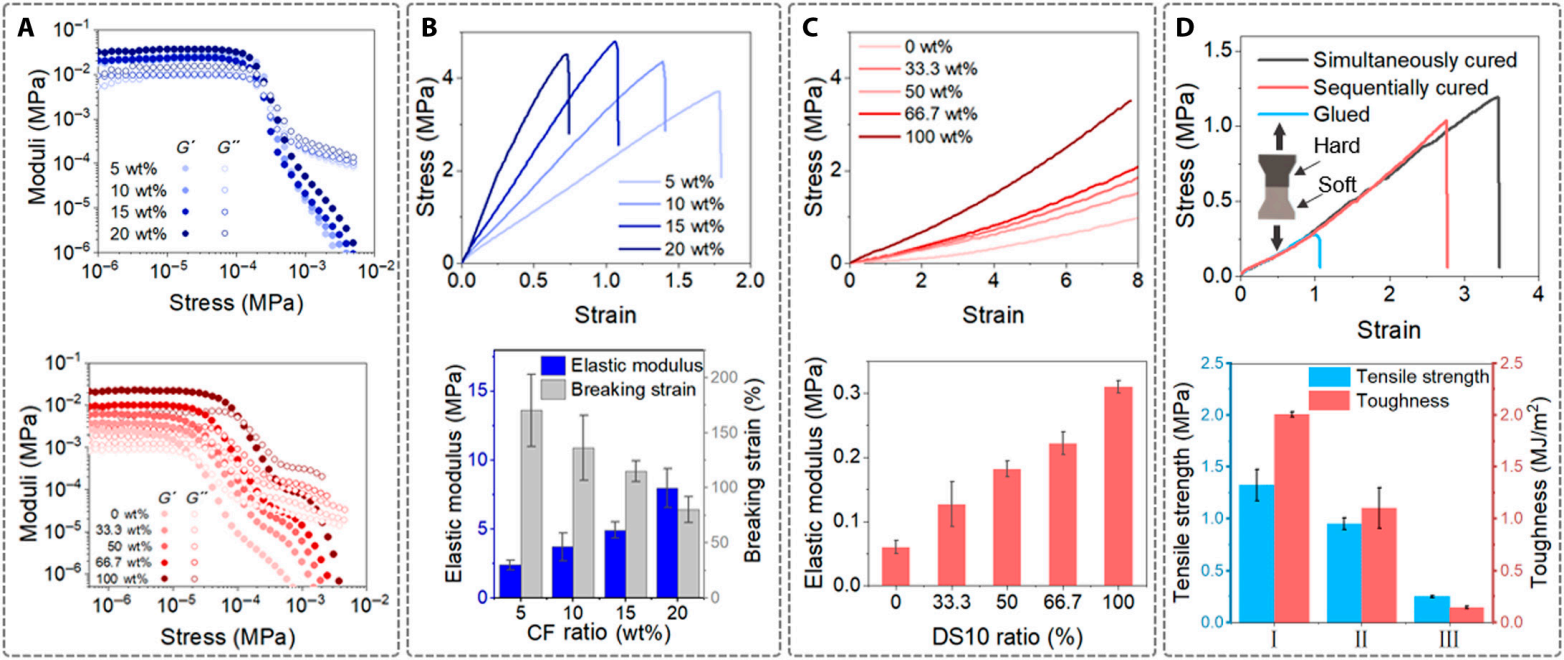
Rheological and mechanical properties of the hard and soft inks. (A) Storage (G′) and loss (G′′) moduli of the hard inks with different concentrations of CFs (top) and soft silicone ink with different mass ratios of DS10 (bottom). (B) Stress–strain curve of the cured PDMS-CF composite inks (top) and their elastic moduli and ultimate elongations (bottom). (C) Stress–strain curves of the cured soft silicone inks and their elastic moduli of cured soft inks (bottom). (D) Stress-strain curves of the dual-material dog-bone shape specimens fabricated by different methods (top) and their tensile strength and toughness of specimens (bottom).

Based on the Voigt model [30], the equivalent elastic modulus *E_eq_* can be predicted by Eq. 1, where *E_h_* and *E_s_* are the elastic moduli of the hard and soft inks, respectively, while *V_h_* is the volume fraction of hard material. To obtain the desired pneumatic responses, *E_h_* should be large enough relative to *E_s_* to ensure that *E_eq_* is far larger than *E_s_* at a given *V_h_*. The soft and hard inks employed here possess elastic moduli of ~0.15 and ~8 MPa, respectively (Fig. 1E). The hard material is composed of Sylgard 184 and SE1700 with short-cut carbon fibers that function as fillers to improve its elastic modulus (Fig. S2). The Sylgard 184+CFs ink viscosity remains within the same order magnitude (from ~2 to ~5 Pa·s) when the concentration of CFs increases from 5 to 20 wt% (Fig. S3), with their loss moduli (*G′′*) always higher than their storage moduli (*G′*) (Fig. S4). The addition of SE1700 (or fumed silica) provides hard inks with shear-thinning properties (Fig. S3). The mixture of SE1700 and Sylgard 184 with a weight ratio of 3:1 is selected as the silicone base of the hard ink [27]. The addition of CF fillers hardly changes its viscosity (Fig. S3) and shear moduli (Fig. 2A, top) but greatly influences its mechanical properties. Importantly, we notice a tradeoff between elastic moduli and ultimate strains of CF reinforced polymer composites, i.e., when the concentration of CFs increases from 5 to 20 wt%, the elastic modulus increases from ~2.4 to ~8 MPa, while the ultimate elongation decreased from ~170% to ~75% (Fig. 2B). This result is consistent with previous studies of the mechanical properties of fiber reinforced silicone rubber materials [31].

$$E_{\mathrm{eq}}=E_{s}\left(1-V_{h}\right)+E_{h}V_{h}$$ (1)

The soft ink should also meet the rheological and mechanical requirements. Several commercially available silicone rubbers (Ecoflex and Dragon skin series) are used to fabricate SPAs [32]. The fully cured Ecoflex 0030 (Eco30) and Dragon Skin 10 (DS10) possess elastic moduli of ~60 and ~300 kPa, respectively. In our work, we mixed Eco30 and DS10 with different ratios to obtain inks with different elastic moduli (Fig. 2C). Meanwhile, the rheological properties of soft inks were modified by THI-VEX (a commercially available thickener). All inks exhibit shear thinning properties (Fig. S5) with *G′* are higher than *G′′* (Fig. 2A, bottom), which are ideal for DIW.

Comparing to the casting method, our method allows the 2 distinct materials to be cured together in a single matrix, potentially allowing stronger adhesion between the 2 materials. Tensile tests of the 3 types of specimens representing different fabrication methods were conducted: (a) Soft and hard materials are cured simultaneously in a mold (ME3P, our method). (b) The soft material is first cured in the mold, followed by pouring hard materials into the mold to cure next (U-axis printing method [21]). (c) Soft and hard materials are cured separately, and they were glued together by Sil-poxy silicone adhesive (casting method). The specimens 1 have the highest tensile strength and toughness (1.32 MPa, 2.00 MJ/m^2^) comparing to specimens 2 (0.95 MPa, 1.10 MJ/m^2^) and specimens 3 (0.25 MPa, 0.14 MJ/m^2^) (Fig. 2D). Interestingly, specimens 1 failed at the soft part while specimens 2 and specimens 3 failed at the interface (Fig. S6), again confirming that our method provides a stronger adhesion at the hard/soft material interfaces.

### Filament morphology and fusion

**Fig. 3.**
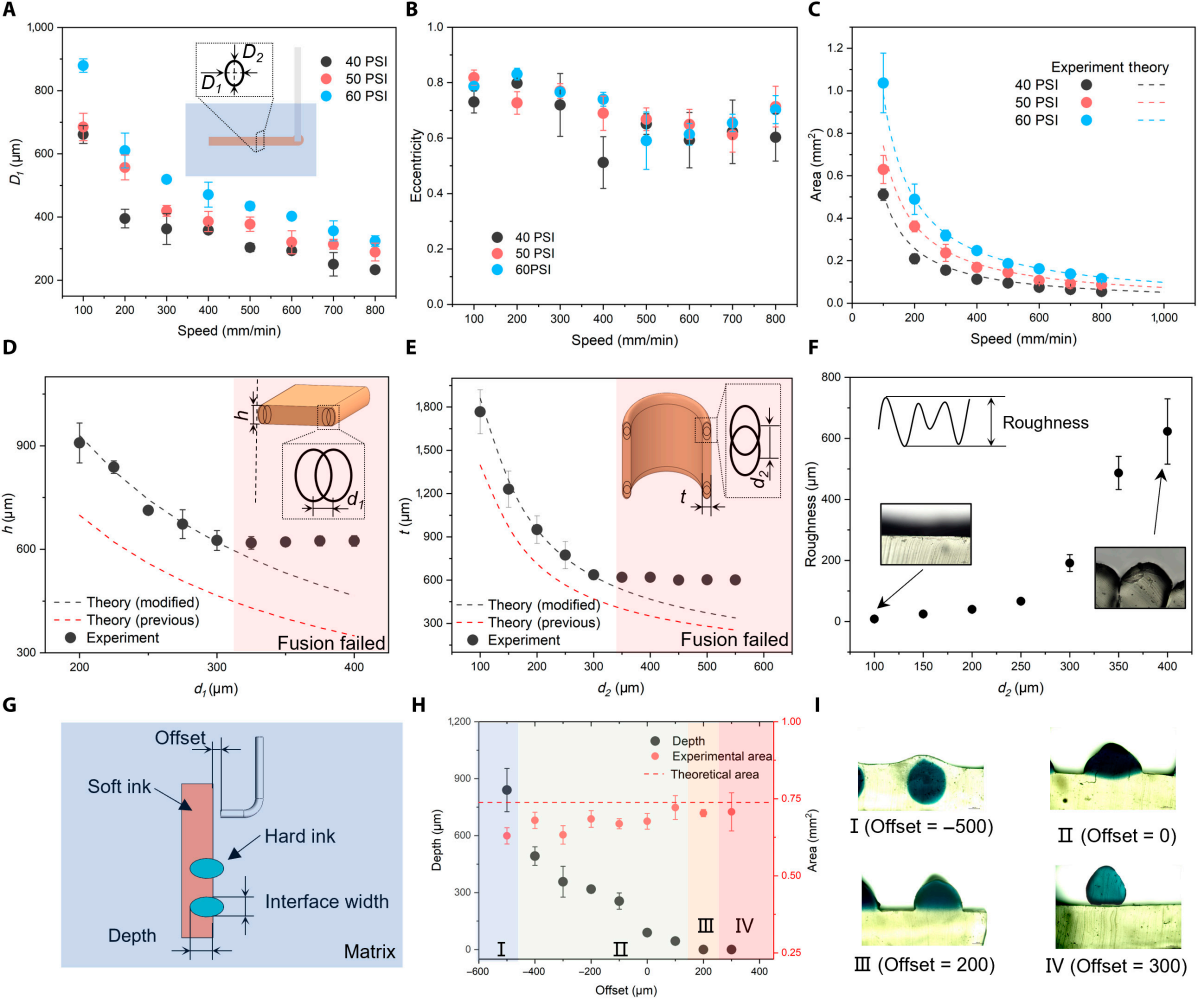
Optimization of embedded 3D printing processes. (A) Impact of the input pressure and nozzle speed on filament diameters. (B) The eccentricity of the cross-sections obtained from the minor and major diameters. (C) Comparisons of filament cross-sectional surfaces measured through experiments (assumed as ellipses) and model predictions. (D) Sheet thickness as a function of step distance in XY direction. (E) Wall thickness of the tube as the function of step distance in the Z direction. (F) Roughness (maximum peak to value height) as a function of step distance in the Z direction. (G) Illustration of conformally printed hard filaments on the surface of printed soft structure. (H) Depth and area of hard fiber as a function of offset. (I) Different relative positions of hard (dark) and soft (transparent) inks when varying the offset values.

Upon choosing the materials family, process parameters were studied by printing filaments in the matrix with different pressures and speeds. Upon curing, the filaments were cut and measured using an optical microscope. Most filaments have an oval-shaped cross-section (Fig. S7). We measured the minor (*D_1_*) and major (*D_2_*) diameters, i.e., the horizontal and vertical diameters of the cross-section. *D_1_* increases with increasing printing pressure and speed (Fig. 3A). We further calculated the eccentricity (*e*) of the cross-section according to *D_1_* and *D_2_* (Eq. 2). When extrusion pressure increases from 275 to 419 kPa and printing speed increases from 100 to 800 mm/min, the eccentricities change from 0.5 to 0.8 (Fig. 3B). Such instability of eccentricity makes the prediction of filament diameter challenging. However, the cross-sectional area can be predicted using Eq. 3 [33,34] (details in Section S3 in supplementary information (SI) and Fig. S8), in which $X=\frac{2\tau_{y}L}{P_{0}R}$, is the ratio of yield and wall shear stress. *R* (0.33 mm) and *L* (38.1 mm) are the radii and lengths of the nozzles; *K_s_* (71.8 Pa·s), *n* (0.712), and *τ_y_* (163 kPa) are the constancy factor, flow index, and yield stress of the ink fitted by the Herschel–Bulkley model, respectively (Fig. S9). *V* is the printing speed. Improving printing pressure and decreasing printing speed increases the cross-sectional area (Fig. 3C). The predicted results match well with the experimental results.

$$e=\sqrt{1-\frac{D_{1}^{2}}{D_{2}^{2}}}$$ (2)

$$\begin{array}{c} \begin{array}{c} S=\frac{\pi R^{3}}{V}{\left(\frac{P_{0}}{2K_{s}L}\right)}^{\frac{1}{n}}\frac{n}{n+1}\\ \left[{\left(1-X\right)}^{\frac{n+1}{n}}-\frac{2n}{2n+1}{\left(1-X\right)}^{\frac{2n+1}{n}}+\frac{2n^{2}}{\left(2n+1\right)\left(3n+1\right)}{\left(1-X\right)}^{\frac{3n+1}{n}}\right] \end{array} \end{array}$$ (3)

The fusion of adjacent filaments guarantees the airtightness of the printed chambers. The degree of filament fusion is determined by the step distance when printing pressure and speed are fixed (344.75 kPa, 400 mm/min). *D_1_* (422 μm) and *D_2_* (561 μm) can be found according to the previous results. We first printed horizontal sheets with different step distances (*d_1_*) to study the filament fusion in the X and Y directions. The sheet thickness (*t_1_*) decreases as the step distance increases from 200 to 300 μm (Fig. 3D). *t_1_* can be predicted based on the volume constant (details in Section S4 in SI). The experimental results match well with the predicted values when *d_1_* is smaller than 300 μm. Otherwise, the fusion fails, leaving gaps between the printed filaments (Fig. S10A). The thickness of the fusion-failed sheet is close to the filaments’ major diameters. Next, we printed a vertical hollow tube to study the fusion in the Z direction. Again, tube thickness (*t_2_*) and step distance (*d_2_*) are inversely proportional when *d_2_* is smaller than 300 μm. Otherwise, the fusion failed (Fig. 3E and Fig. S10B). Comparing to the previous report [35], our theory improves the prediction accuracy by considering the oval-shaped cross-section of the embedded printed filaments rather than the circular shape (Fig. 3D and E). We define a dimensionless $\lambda =\frac{D-d}{D}$ as the overlap of the 2 adjacent filaments. Successful fusion in the X and Y directions requires *λ_XY_* > 0.3, while the successful fusion in the Z direction requires the overlap *λ_Z_* > 0.45. When printing the vertical tube, the first printed filament in the Z direction will be pushed down by the extruded ink at the nozzle tip [25], resulting in *λ_Z_* > *λ_XY_*. The fusion of filaments determines the surface roughness of the printed structure (Fig. S10C). The surface roughness of the printed vertical wall (maximum peak to value height, *R_z_*) increases from ~10 to ~600 μm as the step distance increases from 100 to 400 μm (Fig. 3F). When the step distance is larger than 300 μm, the roughness increases dramatically due to the filament gap of the unsuccessful fusion.

Upon printing the main bodies of CRAs, we next conformally printed reinforced patterns on the surface of the CRAs within the same matrix. We first define a nozzle offset value, which is the distance between the tip of the bent nozzle and the surface of the first printed CRAs (Fig. 3G). To improve the printing accuracy during the multimaterial printing, we first printed a vertical wall in the matrix using the abovementioned process parameters. We next printed filaments on the surface of the printed wall with a bent nozzle (20 G) at a slower speed (100 mm/min). As the offset increases from −500 (negative values indicate insertion of the nozzle into the printed surface) to 300 μm, 4 states of the second printed filament positions are defined (Fig. 3H and I): (a) Offset = −500 μm: the filament is fully embedded in the printed wall; (b) −400 μm ≤ Offset ≤ 100 μm: a part of the filament is embedded into the wall; (c) Offset =200 μm: the filament and wall are interfaced by a straight line; (d) Offset ≥ 300 μm: filament and surface are separate. The depth of the filament decreases from ~840 μm as the nozzle moves away from the surface (Fig. 3H). The insertion depth of the ink is larger than that of the nozzle due to the accumulation of the ink at the nozzle tip in the extrusion direction. The areas of cross-sections of the second printed filaments stabilize at ~0.7 μm^2^, consistent with predictions from Eq. 3.

### Response of printed pneumatic actuators

**Fig. 4.**
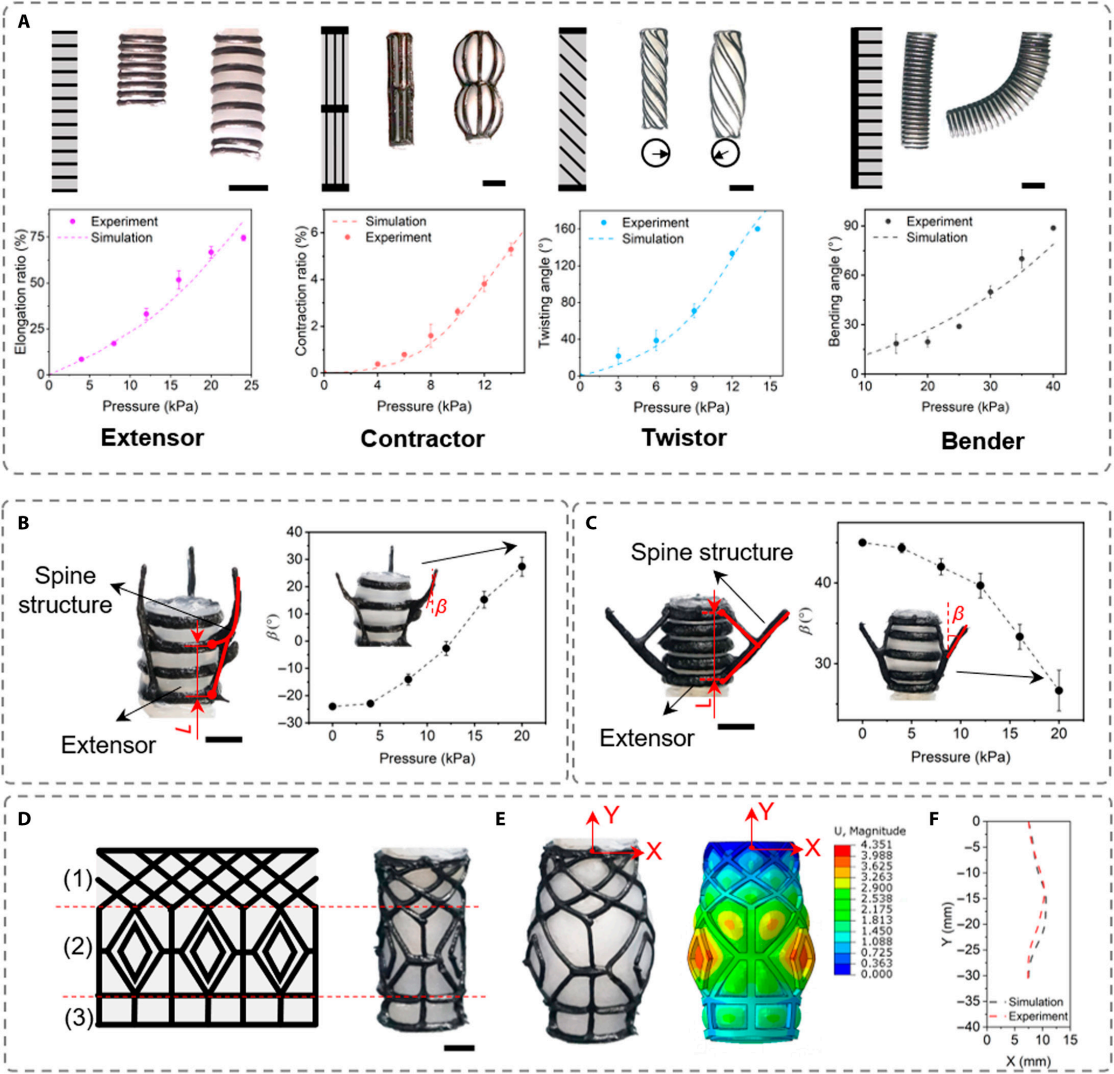
Programmable response of actuators and shape morphing structures. (A) Top: Inflation of the soft elongator (inflating pressure: by 24 kPa, scale bar: 10 mm), contractor (inflating pressure: 14 kPa, scale bar: 10 mm), and twistor (inflating pressure: 14 kPa, scale bar: 10mm) and bender (inflating pressure: 40 kPa, scale bar: 10 mm). Bottom: Experimental and FEA results. The extensors with spine-like structures will open (B) and close (C) upon inflation (scale bar: 5 mm). The dependance of *β* (the angle between the spine tip and vertical line) on inflating pressures are also shown in (B) and (C), right. (D) Printed shape morphing actuator (right, scale bar: 5 mm) with its hard composite pattern (left). (E) The experimental (left) and simulation result (right) of the shape of the tube actuators upon an inflating pressure of 30 kPa. (F) Comparison between the experiment and simulation results of the shape morphing actuator showing its projected profile of its final shape on the right.

Based on the optimized parameters, we then printed the basic actuator units. Programmable pneumatic responses are achieved by tuning the orientation angle (*α*, relative to the longitudinal axis) of the reinforced fibers and geometric parameters of inflating bodies (Fig 4A, top, and Movie S3). When *α* = 90° and the inflating body is in a tubular shape, the actuator elongates ~75% with a pressure of 24 kPa. Contraction is generated as *α* is tuned to 0°. The contraction ratio reaches ~6% with a pressure of ~14 kPa. This actuator can also serve as an expander because of its radial expansion, which is ~200% with a pressure of ~14 kPa. Tuning *α* to the value between 0° and 90° shifts the response from linear to twisting motion. As inflating pressure increases to ~14 kPa, the printed actuator (*α* = 30°) twists ~160°. Adding a 0° constraining fiber at the right side of the elongator, the actuator bends rightwards. The bender bends ~90° with an inflating pressure of ~40 kPa. The inflating processes of 4 actuators are predicted by the FEA (details in Section S2 in SI and Figs. S11 and S12). The simulation results match well with the experimental ones (Fig. 4A, bottom, and Fig. S13). The geometric sizes of the printed actuators are listed in Table S1. We further printed a bender with different scales (Fig. S14). The smallest bender has a wall thickness of 400 μm and a diameter of 4 mm, which is challenging to be fabricated via the traditional casting method. Other features of the abovementioned actuators including the pressure-bearing ability, stiffness, and force are all measured (Figs. S15 to S17). Taking the twistor as an example, thanks to the thin wall thickness and soft inflating materials, the twisting angle of our actuator is one of the highest compared to those fabricated by traditional casting [7,9,12,36 ,37] and U-axis-based printing methods [10,21] (Table S2).

Tuning the orientation angle of the constraining fiber to 15° results in the helical bending motion of the actuator (Fig. S18A), which is controlled by the constraining fiber direction (Fig. S18B). We demonstrate the bioinspired design of cephalopod tentacles’ dexterous movements by changing the inflating body shape to a cone tube (Fig. S19). The reconfigurable response can be achieved by combining different reinforced patterns in a single actuator. In a more complex bending actuator, bending curvature is controlled by the density of horizontal fibers (Fig. S20A), while bending directions can be controlled by the relative positions of the constraining fibers. (Fig. S20B). Thanks to the freeform 3D printing ability, we further demonstrate 2 designs of the embedded 3D printing of spine-like structures on inflating body or reinforced composites to generate local motions. The spines are connected to short ribs printed directly the restricting rings either below (Fig. 4B) or above (Fig. 4C) the spines. Upon inflation, the change in the relative distance (*L*) between the fiber rings will make the spines either expand toward outside or close toward inside, showing an increase and decrease of *β* (the angle between the spine tip and vertical line) from −24° to 27° (Fig. 4B, right) and from 45° to 26° (Fig. 4C, right) with an inflating pressure of 20 kPa, respectively.

In addition, we also print shape morphing actuators to demonstrate the conformal integration of hard materials on the surface of soft inflating body with arbitrary patterns. For demonstration, we print an actuator that will morph from a tubular to a vase-like shape. The hard composite pattern consists of 3 segments from the top to the bottom of the CRA: (a) a woven pattern with a cross angle of ± 45° with the top of the woven constrained by a ring; (b) a periodic lozenge pattern limiting the extensions of the actuator; and (c) a pattern with periodic panes to limit both the extension and the expansion of the actuators (Fig. 4D). The theoretical modeling of the CRA upon its inflation (30 kPa) shows a vase-like shape (Fig. 4E, left) that matches well with the experimental result (Fig. 4E and F).

### Inverse design of the serially connected actuator for point positioning task

**Fig. 5.**
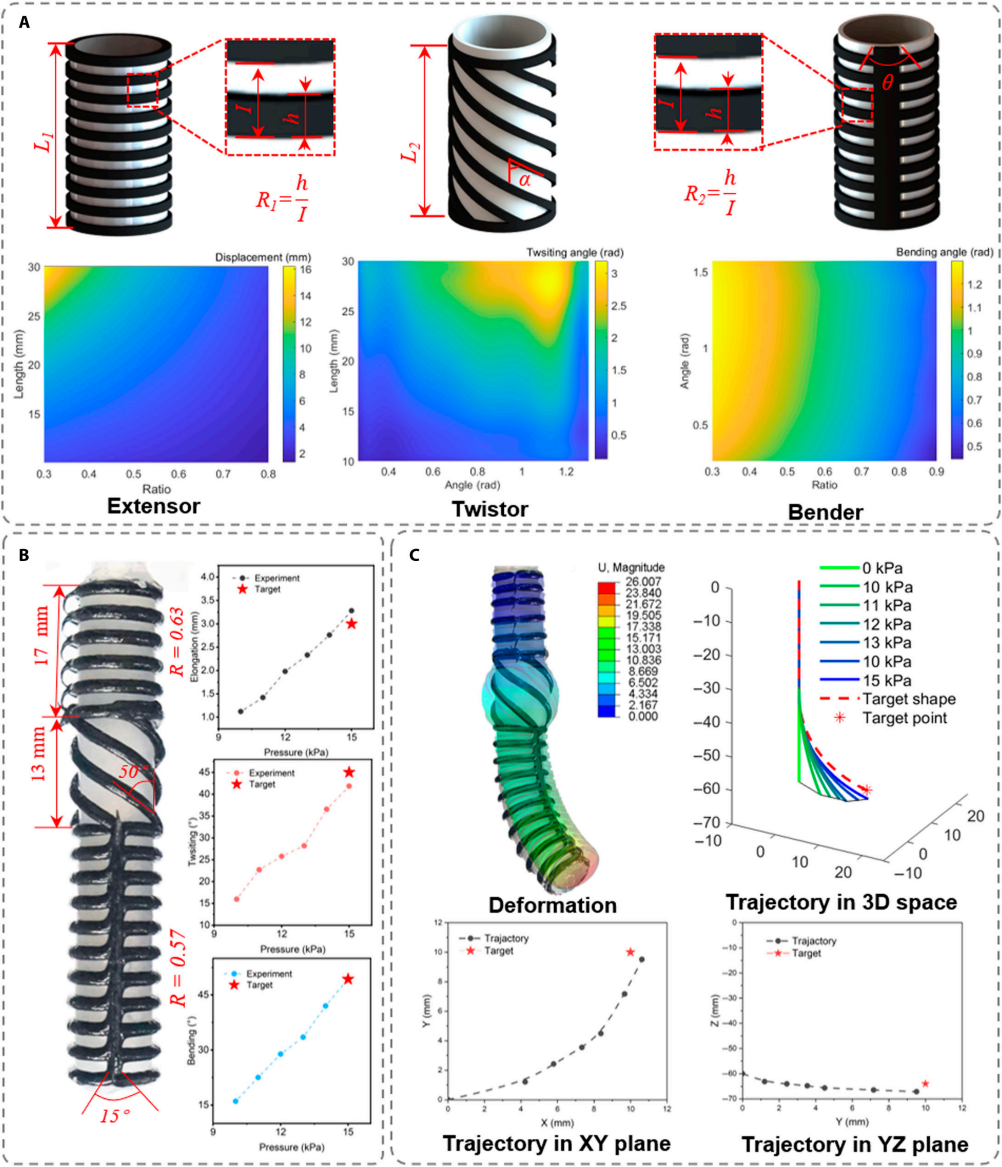
Inverse design of a serially connected actuator for point position task. (A) Top: Design parameters of elongator, twistor, and bender. Bottom: The predicted pneumatic response of 3 actuators with different design parameters. (B) Fabrication parameters of the actuator (left) and the experimental response of elongator, twistor, and bender as the inflating pressures increase from 0 to 15 kPa (right). (C) The experiment (black and white) and FEA inflation of the actuator at the inflating pressure of 15 kPa and the trajectory of the actuator in XYZ space, XY plane, and YZ plane.

Next, to demonstrate the rapid prototyping of complex actuators, as a proof of concept, we employed an FEA-data-based inverse-design method to guide the manufacturing of arbitrary-shaped CRAs with elongator, twistor, and bender as the basic structural units. The responses of each actuator (elongation distance *d*, twisting angle *γ*, and bending angle *θ*) are tuned by 2 parameters (Fig. 5A, top). For simplification, we predefined the length of the desired CRA (*l_total_*, 60 mm) and the length of the bending actuator (*l_bender_*, 30 mm), and arranged 3 actuators in order of elongator, twistor, and bender. The inflating pressure was set as 15 kPa, which is the maximum pressure under which the pneumatic responses are predictable (Fig. S21). The inverse design requires 2 steps of mapping (Fig. S22): (a) mapping Cartesian coordinates (*X*, *Y*, *Z*) to the pneumatic response of actuator units (*d*, *γ*, *θ*); (b) mapping pneumatic response (*d*, *γ*, *θ*) to the design variables (*L_1_*, *R_1_*, *L_2_*, *A_1_*, *A_2_*, *R_2_*). The first mapping is achieved by the inverse kinetic model (Eq. 4, details in Section S5 in SI and Fig. S23). To accomplish the second mapping, we established a surrogate model from design variables to pneumatic response. We employed 2 commercial software (Solidworks for the generation of the geometry of actuators and Abaqus for FEA, Fig. S24) using Python scripts to sample the training data (Fig. S25). The training data were then fitted to the surrogate model using Kriging’s method (details in Section S6 in SI). Using the surrogate model, we can predict the pneumatic response of soft actuators according to the design variables (Fig. 5A, bottom). To minimize the usage of hard materials, we set the optimization target as the minimization of the total volume of reinforced patterns. Therefore, the second mapping is an optimization problem that is described by Eq. 5, where *V* is the total volume of hard ink, and *Srgt_e_*, *Srgt_b_*, and *Srgt_t_* are surrogate models for elongator, bender, and twistor, respectively. We set the point positioning task as the move from (0,0,60) to (10, 10, −64) in an XYZ space. The corresponding pneumatic response of the elongator, twistor, and bender are 3.02 mm, 0.785, and 0.86 rad, respectively. Using the multiobjective optimization algorithm [38] (details in Section S7 in SI), we can obtain the final design variable values for *L_1_*, *R_1_*, *L_2_*, *A_1_*, *A_2_*, and *R_2_* as 16.9 mm, 0.66, 13.1 mm, 0.92 rad, 0.261 rad, and 0.56, respectively. All design variables converged to optimal value after ~40 iterations (Fig. S26).

$$\left\{\begin{array}{l} \;\gamma =\arctan\left(\frac{y_{1}}{x_{1}}\right)\\ \frac{L}{\theta }-\sqrt{\left(x_{1}^{2}+y_{1}^{2}\right)}+6\cos\theta -6=\frac{L}{\theta }\cos\theta \\ d=\Delta z-l_{\text{bending}}-\left(\frac{l_{\text{bending}}}{\theta }+6\right)\sin\theta \end{array}\right.$$ (4)

$$\begin{array}{l} \min:\;V=16.485L_{1}R_{1}+495.55R_{2}+78.75A_{2}\left(1-R_{1}\right)+9.44\left(30-L_{1}\right)/\sin\left(A_{1}\right)\\ s.t.\;:\;{\text{Srgt}}_{e}\left(L_{1},R_{1}\right)=d;{\text{Srgt}}_{b}\left(R_{2},A_{2}\right)=\theta ;{\text{Srgt}}_{t}\left(30-L_{1},A_{1}\right)=\alpha ; \end{array}$$ (5)

The design values were then used to print serially connected actuators (Fig. 5B, left). The actual parameters agree with the designed ones. When the inflating pressure reaches 15 kPa, the elongator section elongates ~3.28 mm, the twistor section twists ~0.729 rad, and the bender section bends ~0.863 rad (Fig. 5B, right). Experimental responses of the actuator (black and white) agree well with the designed shape by FEA (colorful, Fig. 5C, left top). The tip of the actuator travels through a spatial arc and reaches the final point (10.6, 9.5, −67.1) at a pressure of 15 kPa (Fig. S27). The reaching point matches well with the target one (Fig. 5C).

### Printed tube-climbing soft robots

**Fig. 6.**
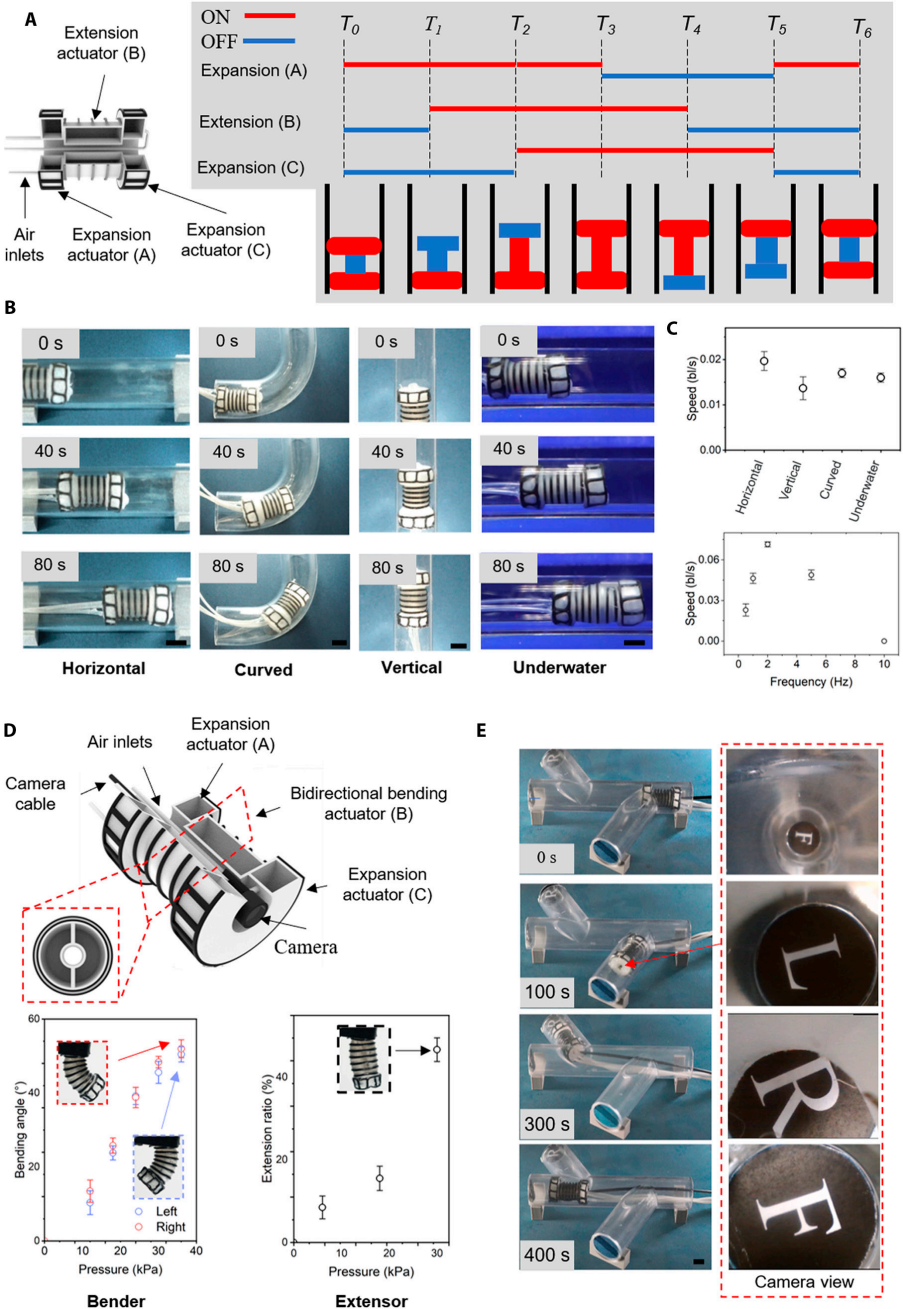
Printed soft tube crawling robots. (A) Structures of the single direction crawling robot (left) and actuation states during the motion sequence (right). (B) The robot climbs through a horizontal, curved, and vertical tube in the air and the tube in the water (scale bar: 10 mm). (C) Top: The speed of the robot crossing different tubes. Bottom: The speed of the robot as a function of inflating/deflating frequency. (D) Top: The improved soft robots can turn into a tube consisting of 2 expansion actuators, a bidirectional bending actuator, and a hosted camera. Bottom: Bending angles of the bidirectional bending actuator at the different inflating pressure of a single air channel and elongation ratios of the bidirectional bending actuator with different inflating pressures of both channels. (E) The robot in (D) crosses a forked tube (left) with a camera in the middle of the robot (scale bar: 10 mm).

As a final demonstration, we printed a soft crawling robot (Movie S4). The robot consists of 2 expanders (*α* = 0°) and 1 elongator (*α* = 90°) (Fig. 6A, left). Periodic inflating and deflating of 3 actuators drives the whole robot to move forward or backward, mimicking the inchworm’s locomotion gait [39]. The inflating pressure is ~30 kPa. The inflating and deflating are defined by ON and OFF states, respectively (Fig. 6A, right). At a cycling frequency of 0.5 Hz, the robot can move across the horizontal, curved, vertical, and underwater tubes at the speed of ~0.02, 0.017, 0.013, and 0.016 bl/s, respectively (Fig. 6B and C and Movies S5 and S6). The speed is also related to the inflating frequency, with an optimized frequency of 2 Hz for a speed of ~ 0.07 bl/s (Fig. 6C, bottom). The speed decreases linearly as the inflating frequency decreases when the inflating frequency is smaller than 2 Hz, which is because actuators fail to expand or extend sufficiently. Despite that the peak speed of our robot (0.07 bl/s) is lower than a recently reported balloon-based structure using vacuum to drive the middle contractor (0.19 bl/s) [40], it is comparable to peak speed of most fluid driven soft crawling robots using the extensor as the middle actuator [41].

We further improved the elongator to a bidirectional bender to provide the crawling robot with turning ability (Fig. 6D, top). A miniature camera is hosted in the middle of the crawling robot for real-time image capturing of the surrounding environment. Inflating either channel of the bidirectional bender can make it bend to the opposite direction while inflating both channels simultaneously extend the actuator axially (Fig. 6D, bottom, and Fig. S28). In our work, we control our robot to move across a forked tube. The real-time images are recorded by the miniature camera (Fig. 5E and Movie S7).

## Conclusion

In this work, we have developed a ME3P method for the facile and programmable manufacturing of CRAs and CRA-based soft robots. Two different inks with tunable elastic moduli are designed to function as the soft inflating body and hard reinforced patterns of CRAs. The rheological properties of inks and process parameters are investigated to optimize the print quality and resolution. We first demonstrate the benefit of our ME3P by printing unit actuators with different pneumatic responses (elongation, contraction, bending, and twisting). Meanwhile, an FEA data-driving method is proposed to design more complex actuators. A multisegment soft actuator is designed and fabricated to demonstrate our design and fabrication method. Two soft tube climbing robots are printed as our final demonstrations. Overall, our method provides a versatile and promising platform for the future production of soft robots.

Although our method of 4-axis embedded 3D printing has demonstrated the improved design freedom for CRAs, we envision that the integration of composite fiber inks conformally at the inner surfaces of the air channels or even within the soft materials could further extend the versatility of the printed CRAs. Regarding material choices, although the CFs filler could improve the mechanical properties of hard ink, its elastic modulus (~8 MPa) is still lower than that of the Kevlar fiber utilized in fiber reinforced actuators. Printable hard inks with better reinforced effects are under development for future applications. In our multimaterial printing, the calibration error of distance between 2 extrusion nozzles could bring the deviations. An automation calibration method and advanced Computer-Aid Design/Manufacturing software are demanded to reduce the error [42,43]. Besides, during the second printing process, the extruded inks may push and distort the first printed structures. Further studies could be conducted about the rheological requirements of inks and matrix for our printing process.

## Materials and Methods

The materials and methods part is presented in Section S1 in Supplementary Materials.

## Data Availability

All relevant data that support the findings are available within this article and the Supplementary Materials.

## Supplementary Materials

Supplementary 1 Section S1. Experimental section. Section S2. Finite element analysis (FEA). Section S3. Estimation of filament cross-section’s area. Section S4. The model between the step distance and thickness of sheet and tubes. Section S5. Inverse kinetic model of a point position task. Section S6. Establishment of the surrogate model (Kriging model). Section S7. Optimization algorithms. Fig. S1. Customed 4-axis printing machine. Fig. S2. Optical photograph of ground carbon fibers (CFs) to improve the mechanical properties of the hard ink. Fig. S3. Viscosity vs. Shear rate of hard ink with and without SE 1700 as the thickener. Fig. S4. Storage and loss moduli of the ink consist of Sylgard 184 and CFs. Fig. S5. Viscosity vs. Shear rate of soft ink composed of Ecoflex 0030 and Dragon Skin 10. Fig. S6. The failed positions of different specimens. Fig. S7. Cross-sections of filaments printed at different printing speed and extrusion pressure. Fig. S8. Geometry and parameters for laminar flow in pipes. Fig. S9. Fitting of Herschel-Bulkley model of printing ink. Fig. S10. Fusion of filaments in different directions. Fig. S11. FEA settings of the bending actuator. Fig. S12. Fitting of the hyperplasticity of soft and hard materials. Fig. S13. FEA simulation of different actuators. Fig. S14. Printed bending actuators in different scale. Fig. S15. Pressure-bearing capability of four actuators. Fig. S16. Stiffness of the actuator. Fig. S17. Blocking force of four actuators. Fig. S18. Helical bending actuators. Fig. S19. Helical bending actuators mimicking the motion of cephalopod tentacles. Fig. S20. Serially connected actuators. Fig. S21. Experiment and simulation responses of the bender. Fig. S22. The projection flowchart from coordinates in Cartesian space to the design parameters. Fig. S23. Illustration of Inverse kinetic model of actuator designed for point positioning task. Fig. S24. Flowchart of our inverse design process. Fig. S25. Sampling data using the FEA method. Fig. S26. The optimization process of the objective function and parameters. Fig. S27. Inflating of the serially connected actuator. Fig. S28. Pneumatic response of bidirectional bending actuator under different inflating conditions. Table S1. Geometry size of actuators and robots. Table S2. Comparison between twistors fabricated by different methods.

Supplementary 2 Movie S1. Printing of the bioinspired flowers. Movie S2. Inflation of the bioinspired flowers. Movie S3. Pneumatic response of printed actuators. Movie S4. Printing of the tube crawling robots. Movie S5. The process of single-direction tube crawling robot crossing different tubes in the air. Movie S6. The process of single-direction robot crossing the tube in the water. Movie S7. The process of tube crawling robot with turning ability crossing the forked tube with camera embedded.
