# Supplementary material for: Multimaterial Embedded 3D Printing of Composite Reinforced Soft Actuators
Source: Research (Wash D C). 2023 Apr 18;6:0122. doi: 10.34133/research.0122 (PMC10202188; doi:10.34133/research.0122)
Supplement: Supplementary 1 — Section S1. Experimental section. Section S2. Finite element analysis (FEA). Section S3. Estimation of filament cross-section’s area. Section S4. The model between the step distance and thickness of sheet and tubes. Section S5. Inverse kinetic model of a point position task. Section S6. Establishment of the surrogate model (Kriging model). Section S7. Optimization algorithms. Fig. S1. Customed 4-axis printing machine. Fig. S2. Optical photograph of ground carbon fibers (CFs) to improve the mechanical properties of the hard ink. Fig. S3. Viscosity vs. Shear rate of hard ink with and without SE 1700 as the thickener. Fig. S4. Storage and loss moduli of the ink consist of Sylgard 184 and CFs. Fig. S5. Viscosity vs. Shear rate of soft ink composed of Ecoflex 0030 and Dragon Skin 10. Fig. S6. The failed positions of different specimens. Fig. S7. Cross-sections of filaments printed at different printing speed and extrusion pressure. Fig. S8. Geometry and parameters for laminar flow in pipes. Fig. S9. Fitting of Herschel-Bulkley model of printing ink. Fig. S10. Fusion of filaments in different directions. Fig. S11. FEA settings of the bending actuator. Fig. S12. Fitting of the hyperplasticity of soft and hard materials. Fig. S13. FEA simulation of different actuators. Fig. S14. Printed bending actuators in different scale. Fig. S15. Pressure-bearing capability of four actuators. Fig. S16. Stiffness of the actuator. Fig. S17. Blocking force of four actuators. Fig. S18. Helical bending actuators. Fig. S19. Helical bending actuators mimicking the motion of cephalopod tentacles. Fig. S20. Serially connected actuators. Fig. S21. Experiment and simulation responses of the bender. Fig. S22. The projection flowchart from coordinates in Cartesian space to the design parameters. Fig. S23. Illustration of Inverse kinetic model of actuator designed for point positioning task. Fig. S24. Flowchart of our inverse design process. Fig. S25. Sampling data using the FEA method. Fig. S26. The [file research.0122.f1.docx]

# Supplementary information:

**Multi-material Embedded 3D Printing of Composite Reinforced Soft Actuators**

**Authors**

Zhenhua Wang^1,3^, Boyu Zhang^2,3^, Qu He^1,3^, Hao Chen^1,3^, Jizhe Wang^2,3^, Yuan Yao^2,3^, Nanjia Zhou^2,3^*, Weicheng Cui^1,3^*

**Affiliations**

1. Key Laboratory of Coastal Environment and Resources of Zhejiang Province, School of Engineering, Westlake University, Hangzhou, Zhejiang Province, China.

2. Research Center for Industries of the Future, and Key Laboratory of 3D Micro/Nano Fabrication and Characterization of Zhejiang Province, School of Engineering, Westlake University, Hangzhou, Zhejiang Province, China.

3. Institute of Advanced Technology, Westlake Institute for Advanced Study, Hangzhou, Zhejiang Province, China.

Correspondence should be addressed to Nanjia Zhou; zhounanjia@westlake.edu.cn, and Firstname2 M2. Lastname2; cuiweicheng@westlake.edu.cn

**S1. Experimental section**

**Printing hardware:** The printing process was finished using a custom-made 4-axis 3D printer (**Fig. S2**). The ink was housed in a syringe (10 mL, EFD inc.) attached by a Luer-lock to a commercial nozzle (20G, Nordson EFD). A straight nozzle was used in the first printing and a 90°bent nozzle was used in the second conformal printing. The printing path was generated via the production of the G-code that outputs the XYZ motion of a custom-made 3D printer. G-code was generated either by hand or using commercial software. Our work used the pressured air regulated by a fluid dispenser (Ultimus V, EFD Inc) to extrude the ink.

**Materials:** Carbomer ETD 2020 was purchased from Lubrizol (U.S.); Sylard 184 and SE 1700 were purchased from DOW CORNING (U.S); Ground carbon fibers were purchased from Tanxi (China); Dragon skin 10, Ecoflex 0030, Slo-JO, THI-VEX were all from Smooth on (U.S.); Silicone rubber adhesive was purchased from Ausbond (China).

**Matrix and ink formulation:** The printing matrix was prepared by adding 0.5 wt% Carbomer ETD 2020 into the DI water. 10M NaOH solution was added to neutralize the solution and formed the carbomer gel. After being degassed in the vacuum, the gel was ready for printing. After the printed structure was cured, we added the 3M HCI solution to liquefy the matrix to pull out the structure. The silicon substrate of the hard ink was prepared by mixing Sylard 184 (A: B=10: 1), and SE 1700 (A: B=10: 1). 5, 10,15,20 wt% of ground carbon fibers (hydrophobized, 500 mesh) were added to improve the mechanical property of hard ink. Soft ink was prepared by mixing Ecoflex 0030 (A: B=1: 1, Smooth on) and Dragon Skin 10 (A: B=1: 1, Smooth on) in the different mass ratios of Ecoflex 0030 (0, 33.3, 50, 66.7, 100 wt%) to tune the elastic modulus. 3 wt% of Slo-Jo (with respect to the total mass of part B) was added to extend the curing time. 1.5 wt% THI-VEX (with respect to the total mass of part B) was added to thicken the uncured silicone. All inks were mixed by a planetary mixer (ARE-310, Thinky Mixer) and then filled into a syringe. After degassing in a centrifugal machine (Neofuge 1600, Heal force), the inks were ready for printing.

**Rheological characterizations:** The rheological properties of the inks were investigated using a controlled stress rheometer (Discovery HR 10, TA Instrument). Ink viscosities were measured as a function of shear rate from 0.01 to 10000 s^-1^. Storage and loss moduli were measured as a function of stress amplitude in the range of 0.1 to 500 Pa at a frequency of 1 Hz. The recovery time of the matrix was measured by transient step shear rate tests. The matrix (carbomer gel) was pre-sheared at a shear rate of 10 s^−1^ for 200 s first and then the shear rate decreased to 0.01 s^−1^. The viscosity variation was recorded during the test.

**Mechanical characterizations:** Dog-bone-shaped tensile test samples (ISO 527-5A) were fabricated. Tensile properties were measured by universal mechanical testing equipment (CTM 6000, Xieqiang Instrument). The force and stiffness were measured by a force gauge (DS2-10, Puyan Instrument).

**Observation of printed structures:** The cross-sections of printed filaments, horizontal sheets, vertical tubes, and other structures were imaged by optical microscopy (SOPTOP, CX400M). The corresponding sizes were measured by NanoMeasurer software (v. 1.2.0).

**Scanning electron microscopy:** The printed flower structure was characterized using a scanning electron microscope (Gemini450, Zeiss, Germany). The cross-sectional pieces were cut from the junction of soft and hard materials. The samples were coated with a 5 nm Pt film before characterization.

**Characterizations of soft actuators:** The actuators were inflated by the pressured air regulated by the proportional valve (SMC, ITV 1030). Pneumatic responses (elongation, contraction, bending, twisting, and trajectory of tip of actuator for point positioning task) were measured in the optical photographs using a customed Matlab code (Matlab R2021b).

**S2. Finite element analysis (FEA)**

The FEA process of the actuators was finished by commercial software (Abaqus 6.14-4, Dassault). The CAD model was first established in another commercial software (Solidworks 2019, Dassault), then saved as the .step file and imported into Abaqus for mesh generation. The soft tube and hard reinforced fibers were modeled separately in Solidworks and then merged in Abaqus. The nonlinearity of the soft and hard materials was considered by applying hyper-elasticity to them. The soft ink was modeled by the 3^rd^ Yeoh model with the coefficients of $C_{10}=3.61\times10^{-2} MPa, C_{20}=5.91\times10^{-4} MPa, \mathrm{and} C_{30}=-2.07\times{10}^{-6} MPa$. The hard link was modeled by the Mooney-Rivlin model with the coefficients $C_{10}=2.52 MPa, \mathrm{and} C_{01}=-1.34 MPa$. We added a small rigid part at the tip of the actuators which hardly deformed under the inflating pressure to indicate the pneumatic response. The rigid material had an elastic modulus of 1 GPa and Poisson’s ratio of 0.45. The mesh type was hybrid 10-node tetrahedral elements (C3D10H). The general static step with Nlgeom was taken in our FEA. Uniform pressure was set on the inner surface of the channel as the loads. An Encastre constraint was enforced on the beginning face of the actuator. The mesh, materials, load, and boundary conditions of bending actuators were shown in **Fig. S3.**

**S3. Estimation of Filament cross-section’s area**

To estimate the filament of the area of the cross-section, several assumptions should first be made:

1. The ink is homogeneous, isotropic, and incompressible. The flow in the nozzle is a steady-state laminar flow.
2. The friction losses are neglected.
3. A no-slip condition is assumed on the surface of the needle.
4. Gravitational force is neglected due to the high pressures applied in the syringe.
5. The ink has the shear-thinning viscosity and yield stress, which can be described by the model (**Eq. S1**), where $\tau$ is the shear stress;$\gamma$ is the shear rate; $K_{s}, n, \mathrm{and} \tau_{y}$ are the apparent viscosity, coefficients of rigidity, and yield stress ^[S1]^.

| $\left\{ \begin{aligned} \tau=\tau_{y}+K_{s}\dot{\gamma}^{n}, \mathrm{for} \left\vert\tau\right\vert>\tau_{y} \\ \dot{\gamma}=0, f\mathrm{or} \left\vert\tau\right\vert<\tau_{y} \end{aligned} \right.$ | (S1) |
| --- | --- |

Due to the yield of the fluid in the pipe, as shown in Fig **S1**, the central part of the fluids will move like as a rigid plug if the shear stress levels are smaller than the yield stress of the fluid. Letting $P_{r}=\frac{\Delta P}{L}$, is the pressure drop per unit length. The shear stress due to the pressure can drop is given by ^[1]^

| $\left\{ \begin{aligned} \tau= \frac{r}{2}P_{r}, \mathrm{for} r>r_{p} \\ \tau= t_{y}, f\mathrm{or} \left\vert\tau\right\vert<\tau_{y} \end{aligned} \right.$ | (S2) |
| --- | --- |

Where $r_{p}$ is the radius at which there is an unsheared portion of the fluid, at which $\tau$ should be equal to $\tau_{y}$. Therefore, $r_{p}$ is given by

| $r_{p}=\frac{2\tau_{y}}{P_{r}}$ | (S3) |
| --- | --- |

According to the flow rate of the Herschel–Bulkley ink (**Eq. S1**) and the shear stress distribution, When $r<r_{p}$, we can get that

| $t_{y}+K_{s}\left( -\frac{du}{dr} \right)^{n}=\frac{r}{2}P_{r}$ | (S4) |
| --- | --- |

the velocity of the flow $u_{f}$

| $u_{f}=\frac{n}{n+1}\frac{2Ks}{P_{r}}\left[ \left( \frac{P_{r}}{2K_{s}}R-\frac{\tau_{y}}{K_{s}} \right)^{\frac{n+1}{n}}-\left( \frac{P_{r}}{2K_{s}}r-\frac{\tau_{y}}{K_{s}} \right)^{\frac{n+1}{n}} \right]$ | (S5) |
| --- | --- |

When $r<r_{p}$, the velocity of the plug $u_{p}$

| $u_{p}=\frac{{n\left( \frac{P_{r}}{2K_{s}} \right)}^{\frac{1}{n}}}{n+1}$ | (S6) |
| --- | --- |

The total flowrate is the integration of velocity

| $Q=2\pi\int_{0}^{r_{p}} u_{p}rdr+2\pi{\int_{r_{p}}^{R} u_{f}rdr=\pi n\frac{1}{K_{s}}}^{\frac{1}{n}}\frac{\left( {P_{r}R}/2-\tau_{y} \right)^{\frac{1}{n}}}{\left( {P_{r}}/2 \right)^{3}}\left[ \frac{\left( {P_{r}R}/2-\tau_{y} \right)^{2}}{1+3n}+\frac{2\tau_{y}({P_{r}R}/2-\tau_{y})}{1+2n}+\frac{\tau_{y}^{2}}{1+n} \right]$ | (S7) |
| --- | --- |

After inducting a dimensionless $X=\frac{2\tau_{y}L}{\Delta PR}$, and some mathematical deformation, we can get the usual form of flowrate of the Herschel–Bulkley fluid in pipe ^[S2]^ .

| $Q=\pi R^{3}\left( \frac{\Delta P}{2K_{S}L} \right)^{\frac{1}{n}}\cdot\frac{n}{n+1}\cdot$  $\left[ \left( 1-X \right)^{\frac{n+1}{n}}-\frac{2n}{2n+1}\left( 1-X \right)^{\frac{2n+1}{n}}+\frac{2n^{2}}{\left( 2n+1 \right)\left( 2n+1 \right)}\left( 1-X \right)^{\frac{3n+1}{n}} \right]$ | (S8) |
| --- | --- |

Where $\Delta P$ is the pressure difference between the inlet and outlet of the nozzle. $L$ and $R$ are the lengths and inner diameters of the nozzle. From mass conservation, Q should be equal to the volume of extruded filament in the matrix, which is

| $Q=V_{p}S_{f}$ | (S9) |
| --- | --- |

Where $V_{p}$ is the printing speed, and $S_{f}$ is the cross-section area. Combined **Eq. S2** and **S3**, we can obtain that

| $S_{f}=\frac{\pi R^{3}}{V_{p}}\left( \frac{\Delta P}{2K_{S}L} \right)^{\frac{1}{n}}\cdot\frac{n}{n+1}\cdot$  $\left[ \left( 1-X \right)^{\frac{n+1}{n}}-\frac{2n}{2n+1}\left( 1-X \right)^{\frac{2n+1}{n}}+\frac{2n^{2}}{\left( 2n+1 \right)\left( 3n+1 \right)}\left( 1-X \right)^{\frac{3n+1}{n}} \right]$ | (S10) |
| --- | --- |

Additionally, if we assume the inlet pressure is the extrusion pressure $P_{0}$ (~300 kPa), and the outlet pressure is the yield stress of the matrix (~0.1 kPa), we can simplify **Eq. S4** to **Eq. S5,** where $X=\frac{2\tau_{y}L}{P_{0}R}$ **.**

| $S_{f}=\frac{\pi R^{3}}{V_{p}}\left( \frac{P_{0}}{2K_{S}L} \right)^{\frac{1}{n}}\cdot\frac{n}{n+1}\cdot$  $\left[ \left( 1-X \right)^{\frac{n+1}{n}}-\frac{2n}{2n+1}\left( 1-X \right)^{\frac{2n+1}{n}}+\frac{2n^{2}}{\left( 2n+1 \right)\left( 3n+1 \right)}\left( 1-X \right)^{\frac{3n+1}{n}} \right]$ | (S11) |
| --- | --- |

**S4. The model between the step distance and thickness of sheet and tubes**

As shown in **Fig. 3D**, the volume of the printed structure ($V$) can be calculated as

| $V=ndLt$ | (S6) |
| --- | --- |

In which $n$ is the number of filaments forming the printed sheet, $d$ is the step distance between adjacent filaments, $t$is the sheet thickness, and $L$is the sheet length. The volume of the sheets can also be calculated based on the number of filaments deposited as follows:

| $V=\frac{1}{4}\pi D_{1}D_{2}nL$ | (S7) |
| --- | --- |

Where $D_{1}$ and $D_{2}$ are the major and minor diameters of the filaments. Combining **Eq. S6** and **S7**, we can get **Eq. S8** which connects the step distance and thickness of the sheet.

| $t=\frac{\pi D_{1}D_{2}}{4d}$ | (S8) |
| --- | --- |

The derivation of the relationship between the thickness of the vertical tube and step distance is similar.

**S5. Inverse kinetic model of a point position task**

The inverse kinetic model is established to project coordinates of (X, Y, Z) to the pneumatic response: elongating displacement ($d$), twist angle ($\gamma$), and bending angle ($\theta$). First, the twisting angle is decoupled as **Eq. S9**.

| $\gamma=arctan(\frac{y_{1}}{x_{1}})$ | (S9) |
| --- | --- |

The bending angle can be calculated with the following assumption:

1. Piecewise constant curvature (PCC) model is taken in our inverse kinetic model.
2. The constraint fiber will just bend but not elongate, i.e., the length of the constraint fiber is constant.

In the twisting plane, the bending angle can be calculated by Eq. **S10-1**.

| $\vert OB\vert=\vert OH_{1}\vert+\vert BH_{1}\vert$ | (S10-1) |
| --- | --- |

In which

| $\vert OB\vert=\frac{L}{\theta}$  $\left\vert OH_{1} \right\vert=\frac{L}{\theta}cos\theta$  $\left\vert BH_{1} \right\vert=\vert AH_{1}\vert+{\vert H}_{1}H_{2}\vert-\left\vert AB \right\vert=\sqrt{\left( x_{1}^{2}+y_{1}^{2} \right)}+r\cos\theta-r$ | (S10-2) |
| --- | --- |

In which $r$ is the outer diameter of the actuator, which equals 6. Combing **Eq. S10-1,2**, we can obtain **Eq. S10-3** to solve the bending angle.

| $\frac{L}{\theta}-\sqrt{\left( x_{1}^{2}+y_{1}^{2} \right)}+6\cos\theta-6=\frac{L}{\theta}cos \theta$ | (S10-3) |
| --- | --- |

Upon the bending angle being calculated, the elongation distance can be calculated by **Eq. S11-1.**

| $d=z_{1}-z_{0}-\Delta z_{bendor}$ | (S11-1) |
| --- | --- |

Where $\Delta z_{bendor}$ is the displacement in the Z direction due to the bending of a bender, which is given by

| $\Delta z_{bendor}=l_{bending}-[(\frac{l_{bending}}{\theta}+6)]sin\theta$ | (S11-2) |
| --- | --- |

Therefore, the elongation distance can be calculated by **Eq. S11-3**.

| $d=z_{1}-z_{0}-[l_{bending}+(\frac{l_{bending}}{\theta}+6)sin\theta]$ | (S11-3) |
| --- | --- |

**S6. Establishment of the surrogate model (Kriging model)**

The Kriging model ^[S3]^ postulates that the output response at a prediction point can be expressed as a linear combination of the output responses of the training points in the neighborhood of the prediction point. The Kriging model can be expressed as:

| $\hat{y}=p\left( x \right)+Z(x)$ | (S12) |
| --- | --- |

Where $p(x)$ represents a polynomial that globally approximates the real response. $Z(x)$is assumed to be the realization of a stochastic process with mean zero and spatial correlation function given by ^[S4]^:

| $COV[Z\left( x_{i} \right),Z\left( x_{j} \right)=\delta^{2}R(x_{i},x_{j})]$ | (S13) |
| --- | --- |

Where $\delta^{2}$ indicates the process variance, and $R(x_{i},x_{j})$ refers to the correlation function between two training points $x_{i}$ and$x_{j}$. Usually, the Gaussian function is used as the correlation function, that is:

| $R\left( \theta\right)=\prod_{k=1}^{Dim} exp(-\theta_{k}d_{k}^{2})$ | (S14) |
| --- | --- |

Where $Dim$ indicates the number of decision variables, $d_{k}$is the Euclidean distance between$x_{i}$ and$x_{j}$, and $\theta_{k}$represents the unknown parameter vector to be determined. Then the predicted value $\hat{y}$at the prediction point $x$ is calculated as:

| $\hat{y}=\hat{\beta}+r^{T}\left( x \right)R^{-1}(y-\hat{\beta}p)$ | (S15) |
| --- | --- |

Where $r^{T}\left( x \right)$ indicates the correlation vector of length $N$ between $x$ and the training points, $y$is the real responses at the training points. The scalar $\hat{\beta}$ and $r^{T}\left( x \right)$ can be expressed as:

| $\hat{\beta}=\frac{(p^{T}R^{-1}p)^{-1}p^{T}R^{-1}y}{N}$ | (S16-1) |
| --- | --- |
| $r^{T}(x)=[R(x,x_{1}),R(x,x_{2}),...,R(x,x_{N})]^{T}$ | (S16-2) |

The variance of the output model is estimated as:

| $\hat{\sigma}^{2}=\frac{(y-\hat{\beta}p)^{T}R^{-1}(y-\hat{\beta}p)}{N}$ | (S17) |
| --- | --- |

The unknown parameters $\theta_{k}$ can be estimated by solving a constrained maximization problem:

| $\max:\Phi\left( \theta\right)=\frac{-[N\ln(\hat{\sigma}^{2})+\ln\left\vert R \right\vert]}{2}$ $s.t.:\theta>0$ | (S18) |
| --- | --- |

In this work, we use a MATLAB® SURROGATES Toolbox developed by Viana (2002) ^[S5]^ to implement the Kriging model.

**S7. Optimization algorithms**

The equilibrium optimizer is inspired by the control volume mass balance model, which is applied to the estimation of dynamic and equilibrium states ^[S6]^. In an equilibrium optimizer, each individual (solution) with its concentration $C$ (position) is regarded as a search agent. In EO, each individual in the population is similar to a solution and the individual's concentration is similar to a particle's position in the particle swarm optimization algorithm. The position updating formulation of EO is as follows ^[S7]^:

| $C=C_{e}+(C-C_{e}).F+\frac{G}{\lambda V}(1-F)$ | (S17) |
| --- | --- |

Where $V$ is defined as a unit, $C_{e}$ refers to the equilibrium candidate, $F$ and $G$ represent exponential term and generation rate respectively. And $\lambda=(\lambda_{1},\lambda_{2},...,\lambda_{n})^{T}$ is a random vector in the interval of [0, 1], $n$ is the number of dimensions of the individual’s concentration $C$.

The equilibrium state indicates the final convergence state of EO. At the beginning of the search process, there is no knowledge about the final equilibrium state, and the equilibrium candidate $C_{e}$ is used to provide a search guide for individuals in the population. In an equilibrium optimizer, equilibrium candidates are defined by the four best individuals selected according to their fitness value during the whole optimization process and an individual whose concentration is the average of the above four best individuals. The equilibrium pool consists of five individuals.

| $C_{e,pool}=\{C_{e(1)},C_{e(2)},C_{e(3)},C_{e(4)},C_{e(ave)}\}$ | (S18) |
| --- | --- |

The concentration updating rule is mainly controlled by the exponential term $F$.

| $F=e^{-\lambda(t-t_{0})}$ | (S19-1) |
| --- | --- |
| $t=(1-\frac{iter}{IT})^{(a_{2}\frac{iter}{IT})}$ | (S19-2) |

Where $t$ is the function of iterations, $t$ decreases with several iterations, $iter,$ and $IT$ represent the current iteration and the maximum iteration respectively. $a_{2}$ is a constant value that controls the exploitation ability of EO. To achieve high convergence by slowing down the search speed, $t_{0}$ is defined as:

| $t_{0}=\frac{1}{\lambda}\ln(-a_{1}sign(r_{0}-0.5)[1-e^{-\lambda t}])+t$ | (S20) |
| --- | --- |

Where, $a_{1}$ is a constant value that affects the exploration ability, $sign(r_{0}-0.5)$ is applied to control the direction of exploration and exploitation, $r_{0}$ is a random number in [0,1]. In this work, the values of $a_{1}$ and $a_{2}$ are set to 2 and 1 respectively, the selection of the two values is consistent with the original EO algorithm. Therefore, the exponential term $F$ can be formulated as:

| $F=a_{1}sign(r_{0}-0.5)(e^{-\lambda t}-1)$ | (S21) |
| --- | --- |

Generation rate plays an important role in the equilibrium algorithm, it is used to improve the exploitation ability of EO.

| $G=G_{0}e^{-\kappa(t-t_{0})}$ | (S22-1) |
| --- | --- |
| $G_{0}=GCP(C_{e}-\lambda C)$ | (S22-2) |
| $GCP=\left\{ \begin{matrix} 0.5r_{1} r_{2}\geq GP \\ 0 r_{2}<GP \end{matrix} \right.$ | (S22-3) |

Where $G_{0}$ represents the initial value.$GCP$is the generation rate control probability. $GP$ represents the generation probability, which is set to 0.5 according to the original EO algorithm. $r_{1}$and $r_{2}$ are two random numbers in [0,1]. $\kappa$ indicates the decay vector. This study assumes $\kappa=\lambda$. Thus, the generation rate can be formulated as:

| $F=a_{1}sign(r_{0}-0.5)(e^{-\lambda t}-1)$ | (S23) |
| --- | --- |

The pseudo-code of the EO optimizer is shown in Algorithm 1.


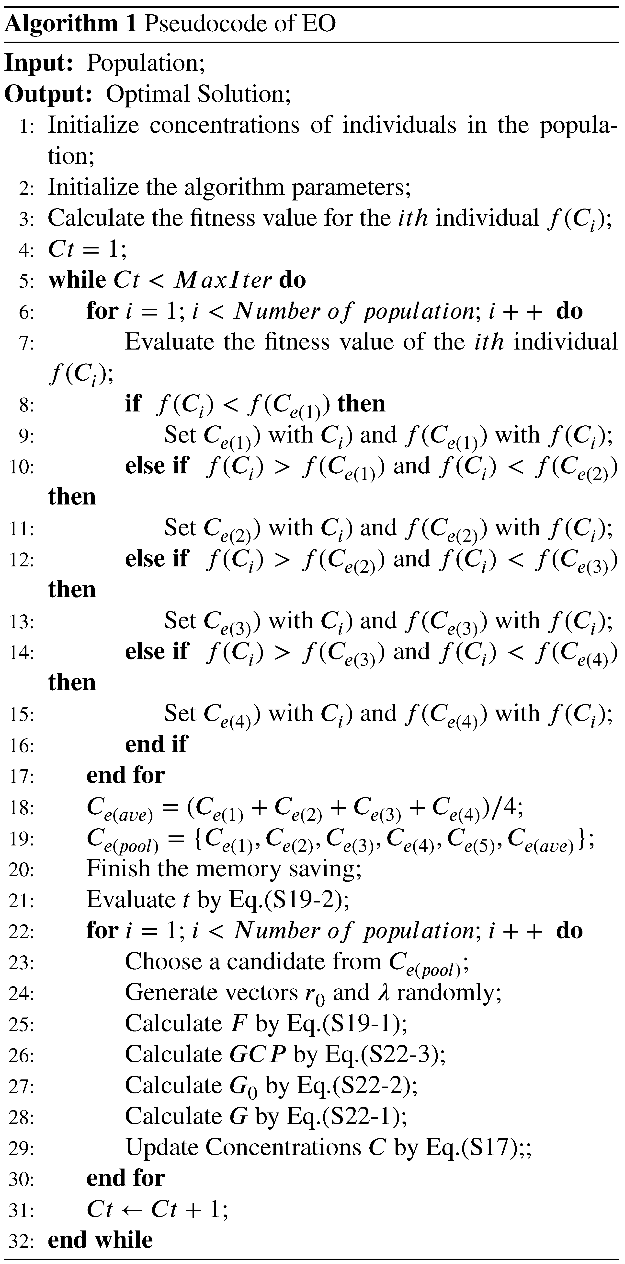


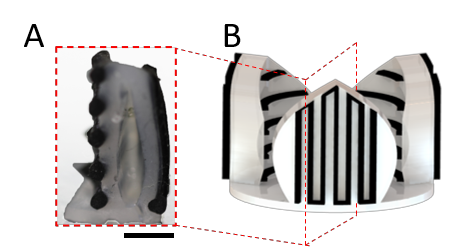


**Fig. S1 Printed bioinspired flower structure:** (A) The air channel in each petal of the structure (Scale bar: 5 mm); (B) The printed flower has three petals, and the vertical fibers on the out cylindrical surface limit the stretch in z-direction while horizontal fibers on the inner cylindrical surface allow the stretch. Thus, the flower will bloom at a certain inflating pressure.


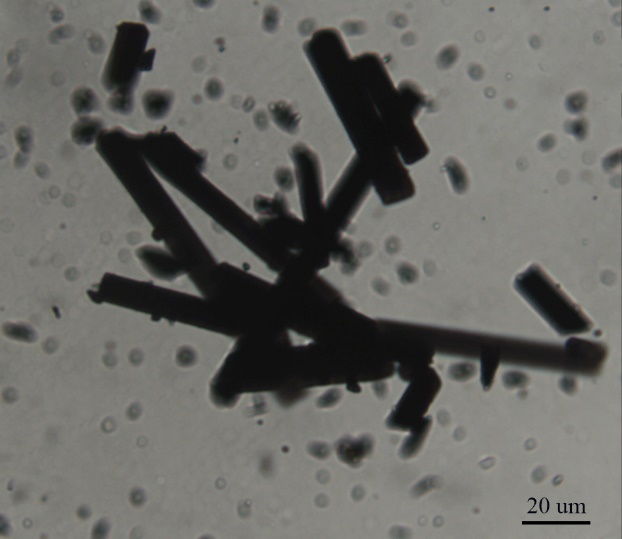


**Fig. S2 Optical photograph of ground carbon fibers (CFs) to improve the mechanical properties of the hard ink:** The CFs have a length of about ~50 $\mu m$ and a diameter of about ~7 $\mu m$.


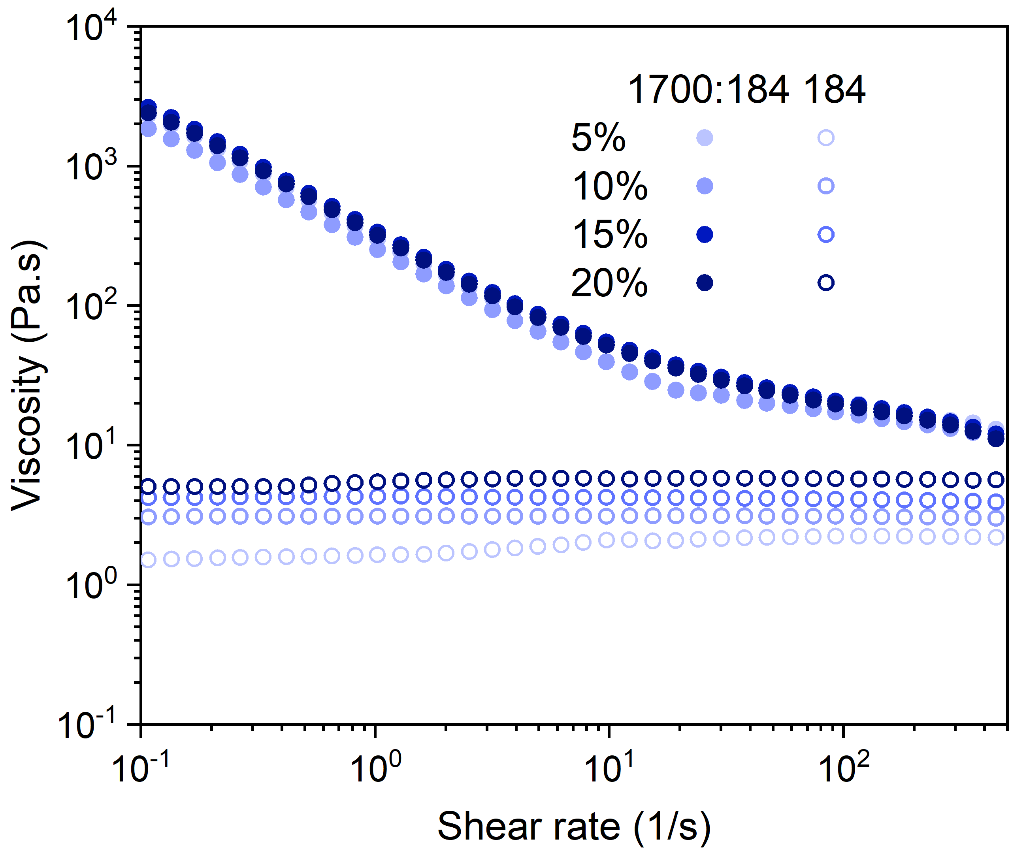


**Fig. S3 Viscosity vs. Shear rate of hard ink with and without SE 1700 as the thickener：** Without SE 1700, the viscosity of the ink hardly changes with the increase of shear rate regardless of the mass ratio of CFs. However, when 33 wt% of SE 1700 with respect to Sylgard 184 is added, the viscosity of the ink decreases as the shear rate increases, which is suitable for direct ink writing.

**
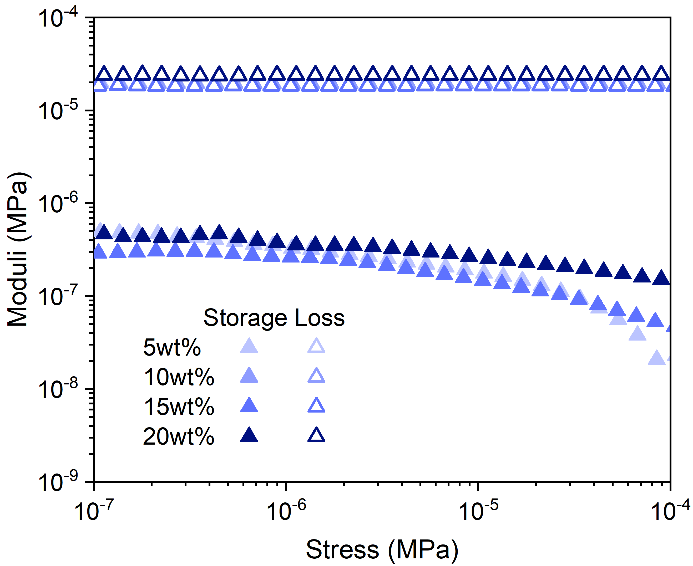
**

**Fig. S4** **Storage and loss moduli of the ink consist of Sylgard 184 and CFs：** As the mass ratio of CFs increases from 5 wt% to 20 wt%, the storage modulus of the ink is usually lower than the loss modulus, indicating the ink is not suitable for direct ink writing.


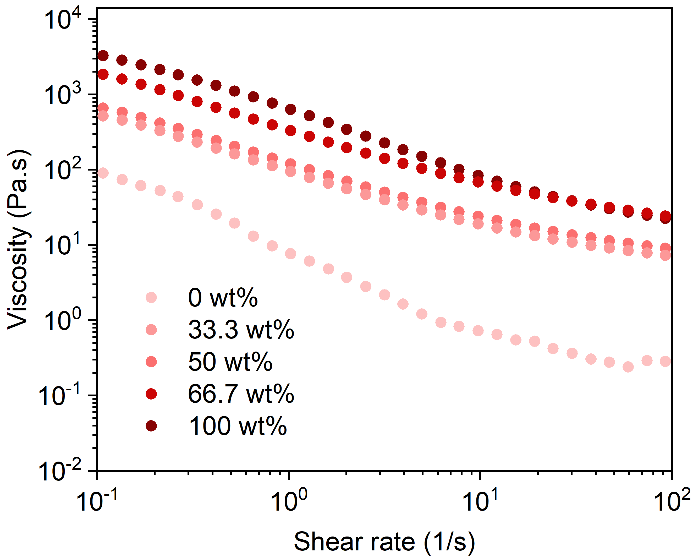


**Fig. S5 Viscosity vs. Shear rate of soft ink composed of Ecoflex 0030 and Dragon Skin 10：** After the rheological modification of soft ink using retarder (Slo-Jo) and thickener (THI-VEX), the soft ink with a mass ratio of Dragon from 0 to 100% all have the shear-thinning viscosity.


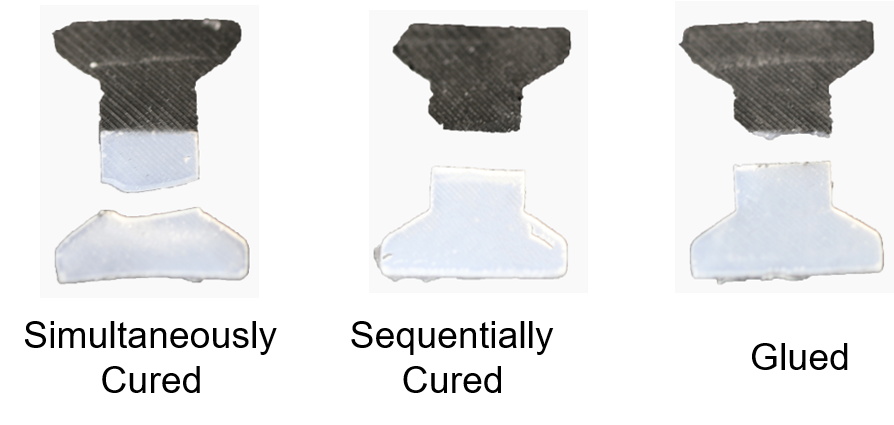


**Fig. S6 The failed positions of different specimens:** The simultaneously cured specimen fails at the soft part while sequentially cured and glued specimens fail at the interface. It indicates that our method provides a stronger adhesion at the interface.


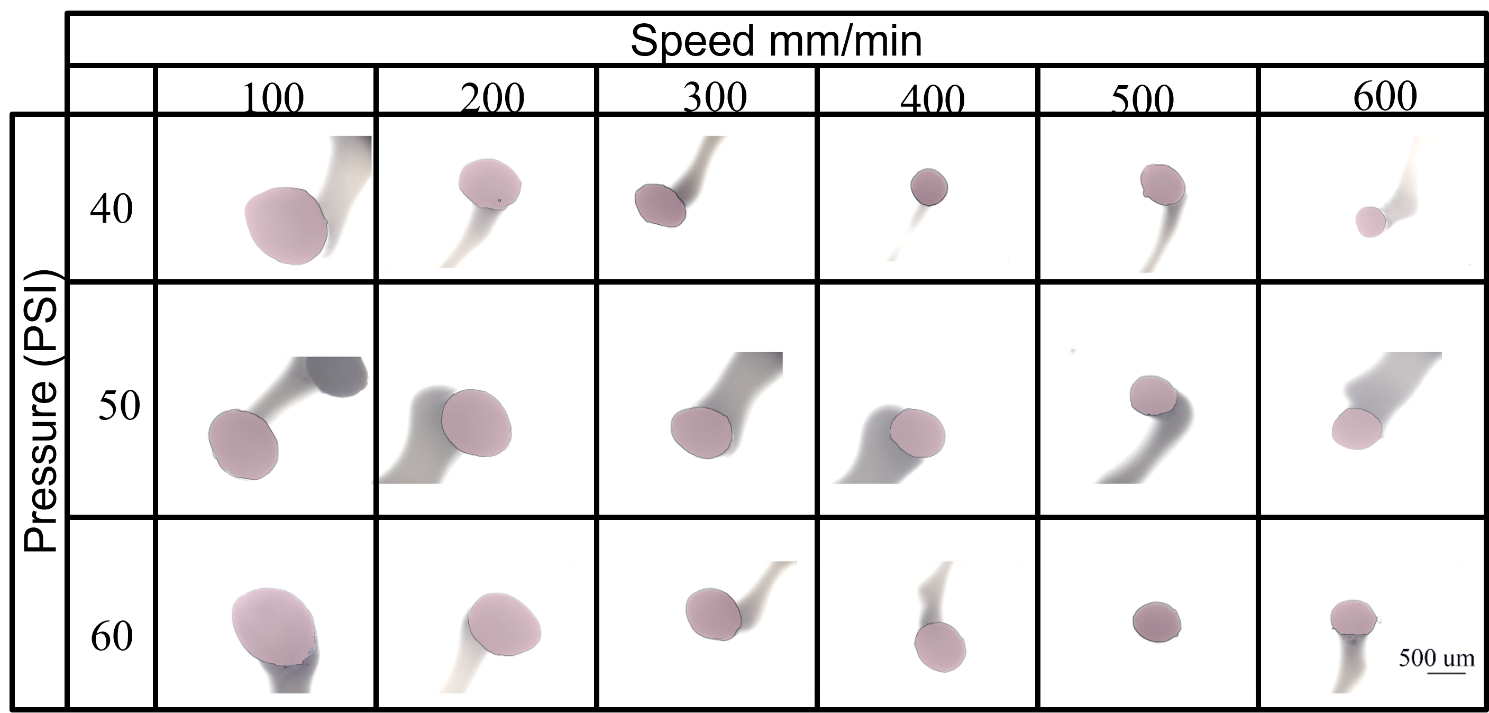


**Fig. S7 Cross-sections of filaments printed at different printing speed and extrusion pressure:** All filaments have oval sections, and the overall size decrease with the increase of printing speed while the decrease of extrusion pressure.


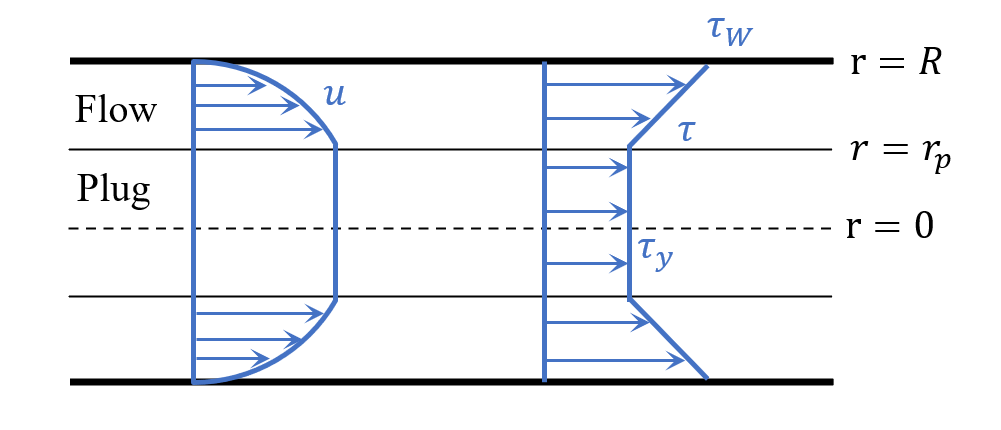


**Fig. S8** **Geometry and parameters for laminar flow in pipes.**

X=(2τ_y L)/∆PR


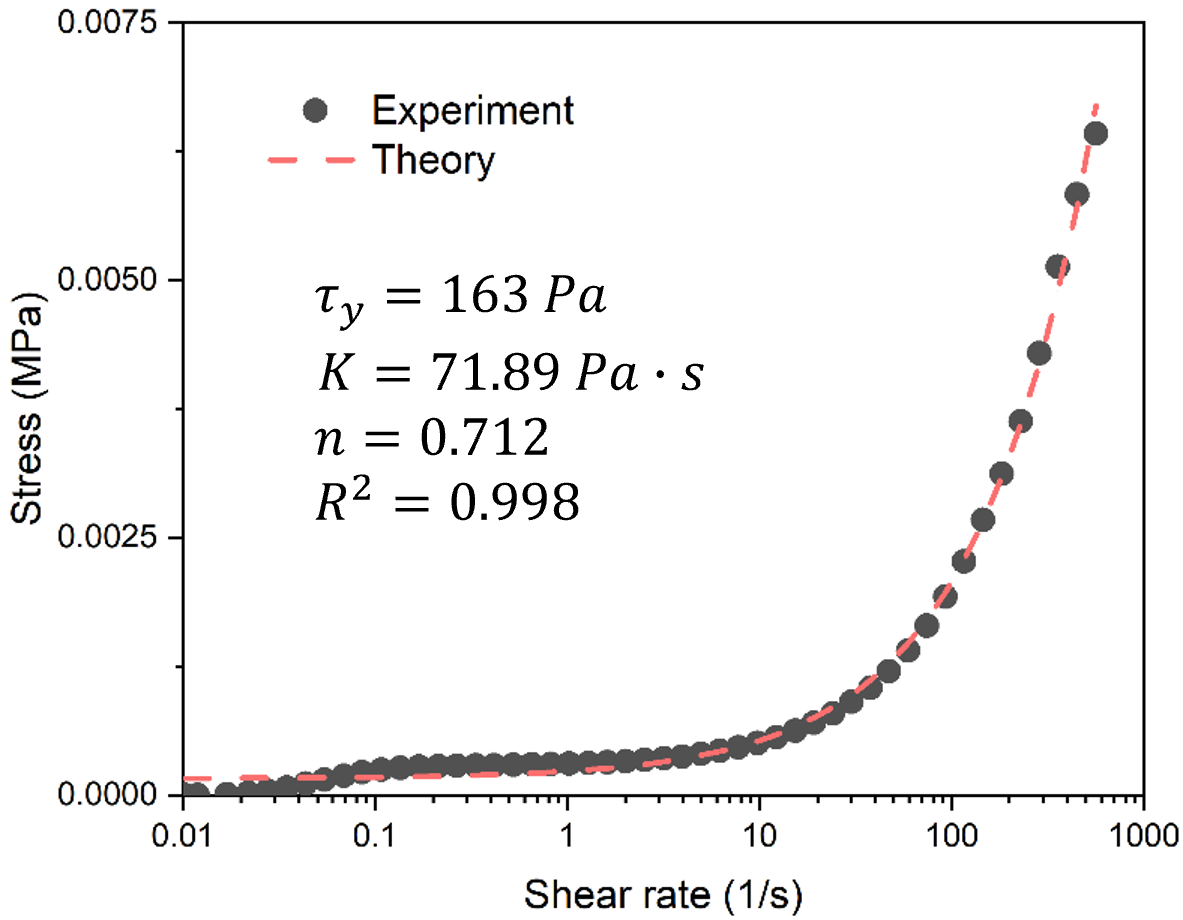


**Fig. S9 Fitting of Herschel-Bulkley model of printing ink:** The coefficients of the ink are fitted as *K_s_* = 71.8 $Pa\cdot s$, *n* = 0.712, and $\tau_{y}$ = 163 kPa.


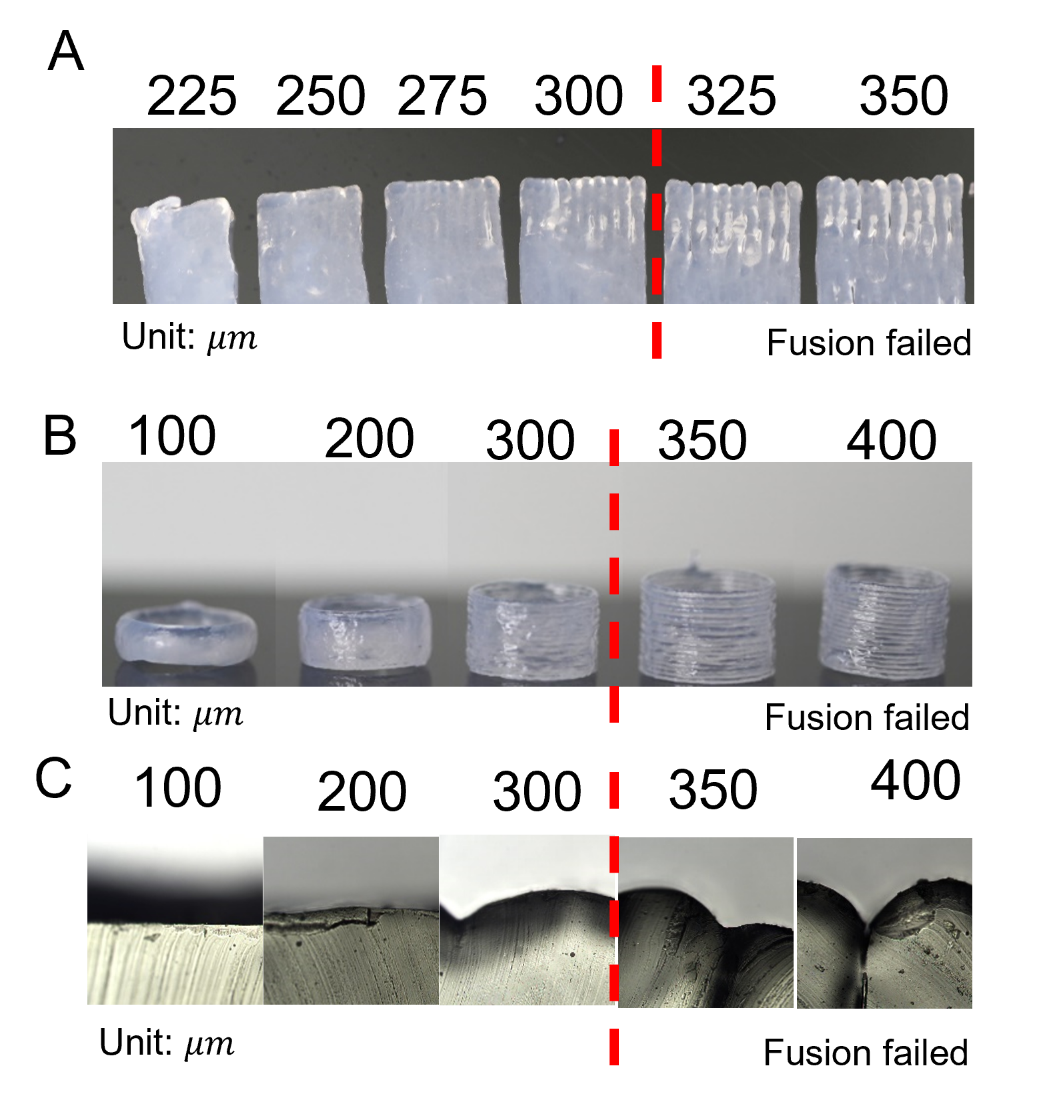


**Fig. S10 Fusion of filaments in different directions:** (A) When the step distance of printed sheets is horizontal < 300 μm, the adjacent filaments fuse well. Otherwise, fusion fails. (B) When the step distance of printed tubes is horizontal < 300 μm, the adjacent filaments fuse well. Otherwise, fusion fails. (C) The surface printed tube becomes rougher as the step distance increases. When the step distance is larger than 350 μm, some visible gaps can be observed between adjacent filaments, indicating failed fusion.


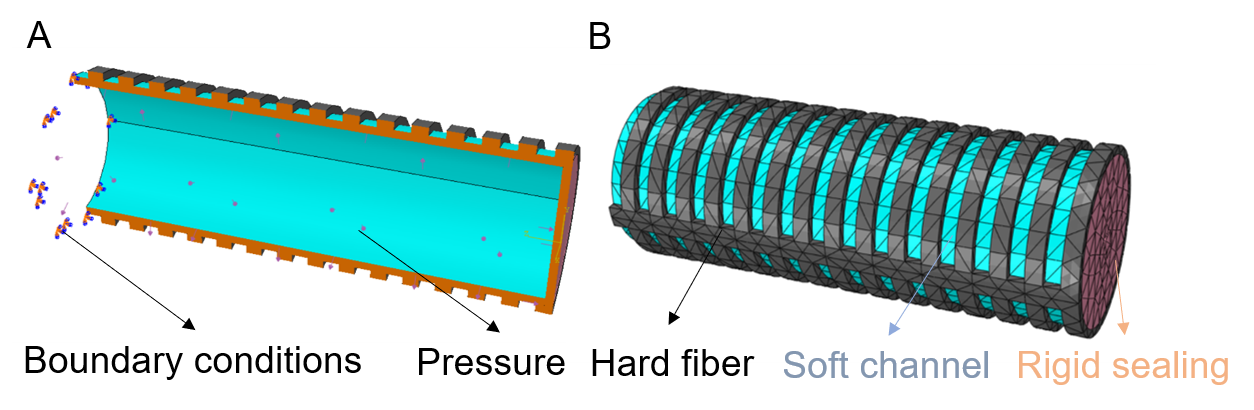


**Fig. S11 FEA settings of the bending actuator:** (A) A Encastre constraint is enforced on the left end. Uniform pressure is set on the inner surface of the channel as the loads. (B) The bending actuator can be divided into three parts: hard fiber (black), soft channel (blue), and rigid sealing (pink). The mesh is assigned by C3D10H type.


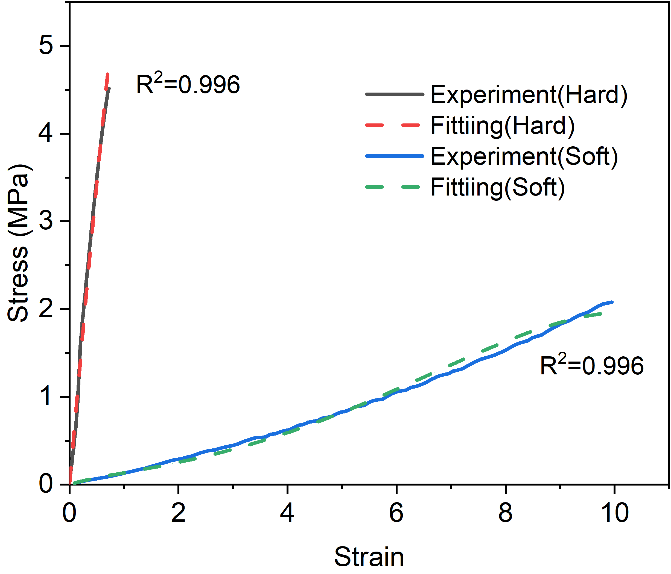


**Fig. S12 Fitting of the hyperplasticity of soft and hard materials:** The soft material is modeled by the 3^rd^ Yeoh model ($C_{10}=3.61\times10^{-2} MPa, C_{20}=5.91\times10^{-4} MPa, \mathrm{and} C_{30}=-2.07\times{10}^{-6} MPa$). The hard material is modeled by the Mooney-Rivlin model ($C_{10}=2.52 MPa, \mathrm{and} C_{01}=-1.34 MPa$).


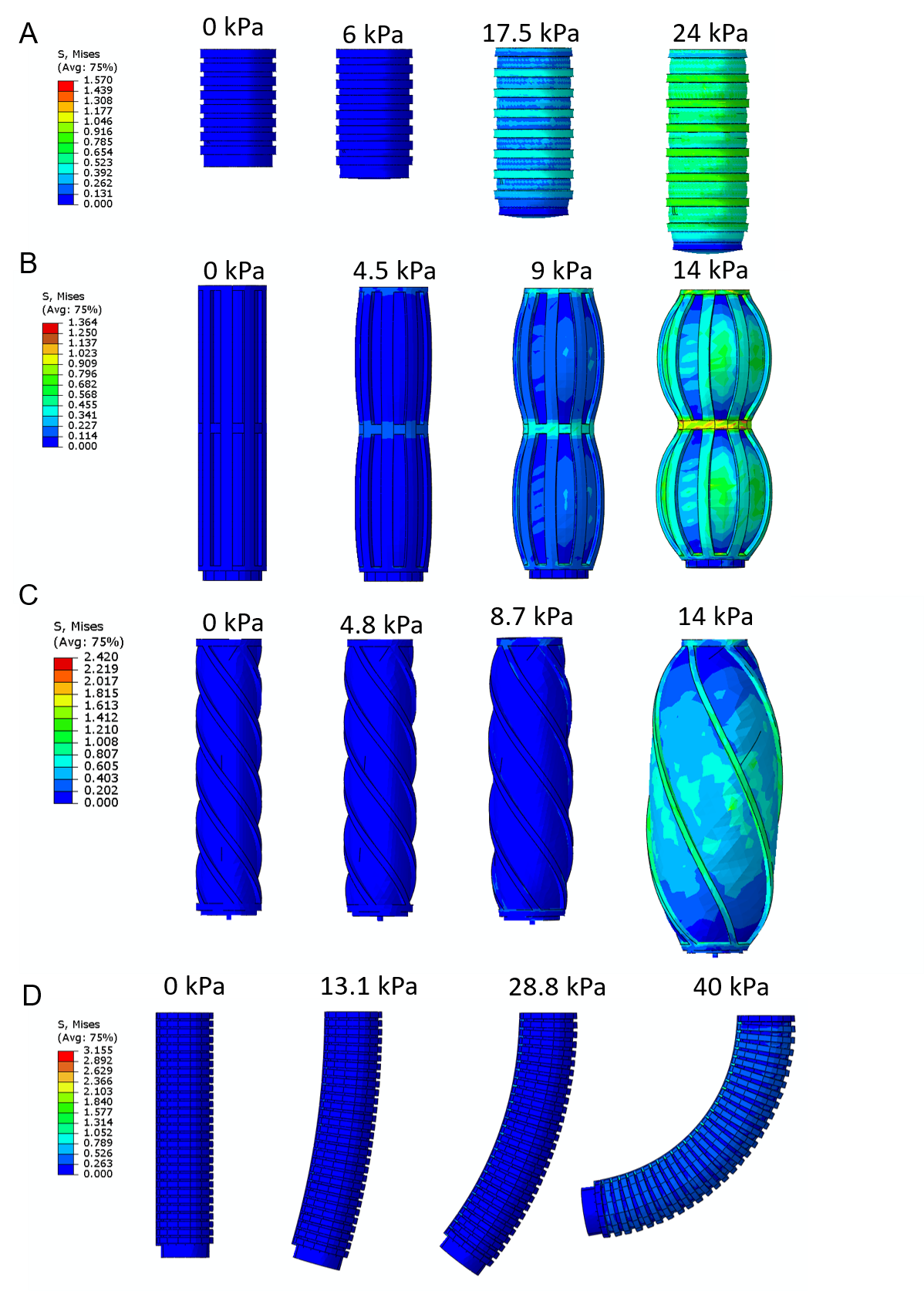


**Fig. S13 FEA simulation of different actuators:** (A) Elongator; (B) Contractor; (C) Twistor; (D) Bender. FEA results match well with the experimental.


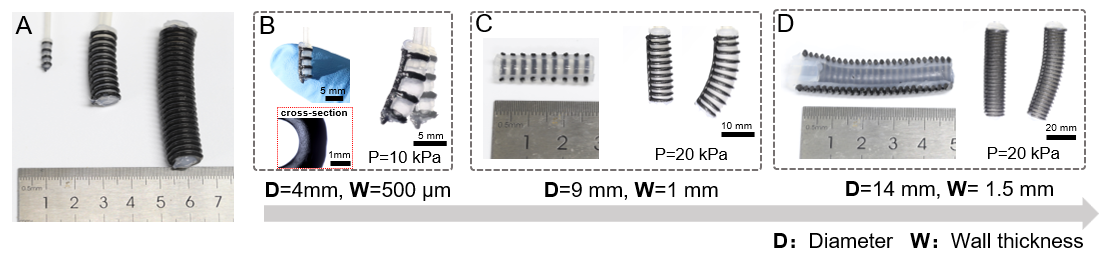


**Figure S14** **Printed bending actuators in different scale:** (A) Comparison between three bending actuators. (B) The bender with a diameter of 4mm and a wall thickness of 500 μm (left) and its bending motion with the inflating pressure of 10 kPa (right); (C) The bender with a diameter of 9mm and a wall thickness of 1 mm (left) and its bending motion with the inflating pressure of 20 kPa (right); (D) The bender with a diameter of 14 mm and a wall thickness of 1.5 mm (left) and its bending motion with the inflating pressure of 20 kPa (right);


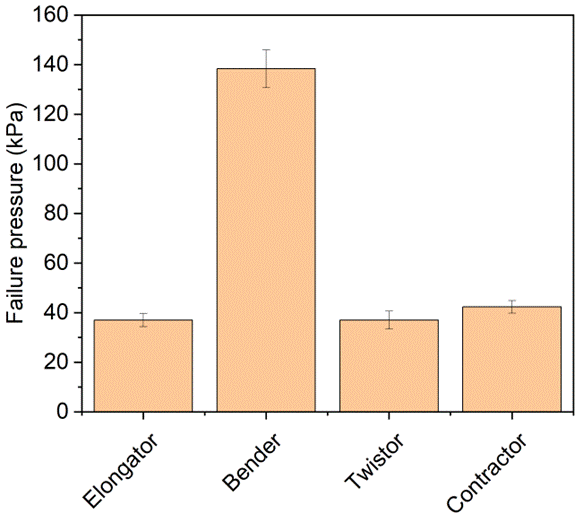


**Fig. S15 Pressure-bearing capability of four actuators:** The elongator, twistor, and contractor have the failure pressure of ~40 kPa. The bender possesses a thicker wall and more reinforced composites, and as a result, its failure pressure is ~140 kPa.


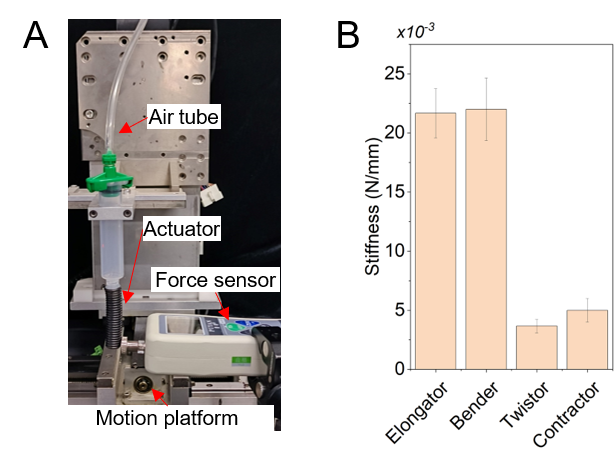


**Figure S16 Stiffness of the actuator:** (A) The measurement platform of stiffness consists of the motion platform, force sensor, and air supply. (B) The elongator and bender has the stiffness of ~0.02 N/mm. The twistor and contractor has the stiffness of ~ 0.005 N/mm. The elongator has the higher stiffness because of the short length while the bender because of the thicker wall and more reinforced composites.


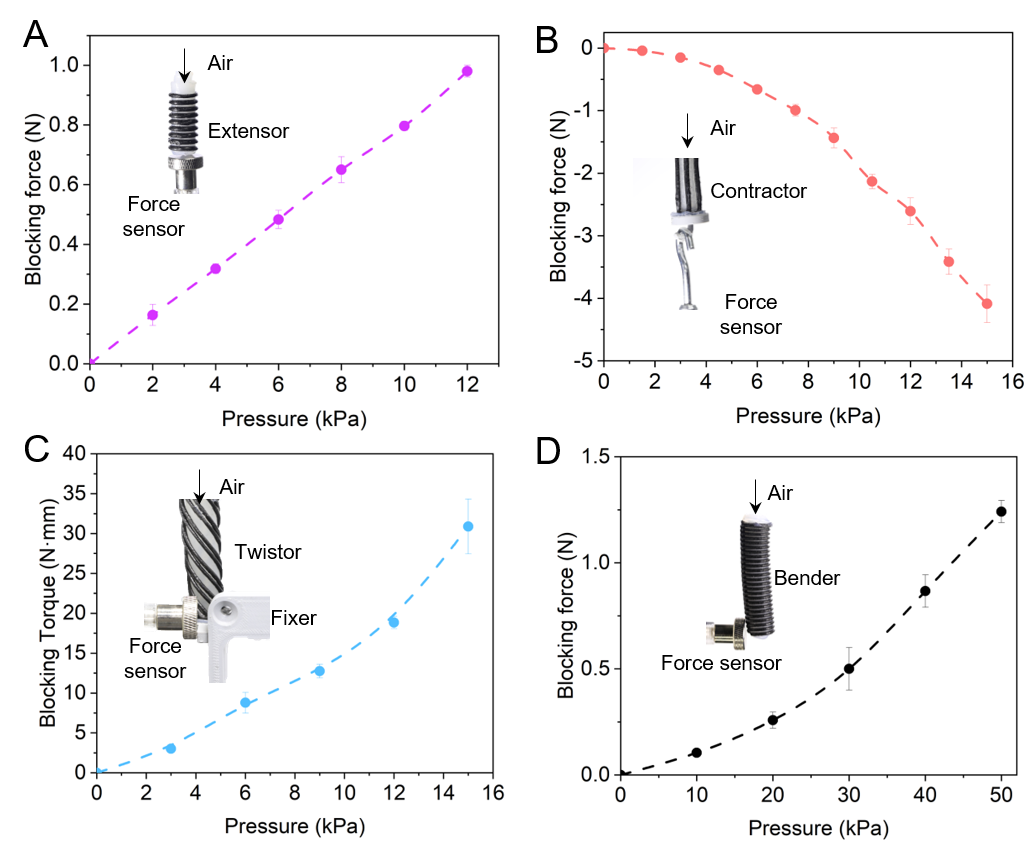


**Figure S17 Blocking force of four actuators:** The blocking force of the extensor, contractor, twistor, and bender are 0.98N, 4.08N, 30N∙mm, and 1.24 N, respectively.


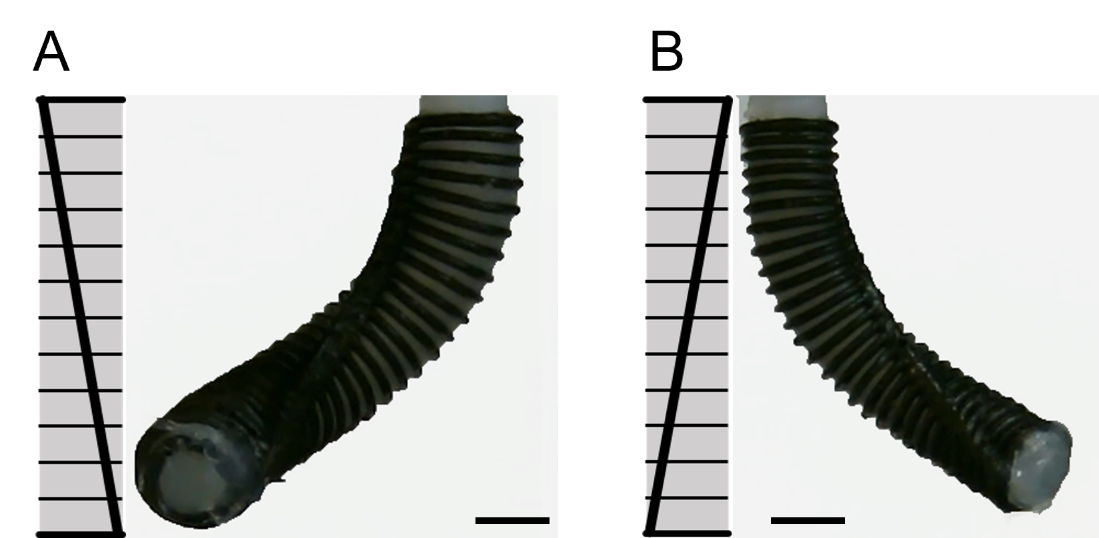


**Fig. S18** **Helical bending actuators:** (A) The helical bending pneumatic response can be achieved by a 15° constraint fiber on the elongator (Scale bar: 10mm). (B) By changing the constraint fiber angle from 15° to -15°, the helical direction is changed from levorotation to dextrorotation (Scale bar: 10mm).


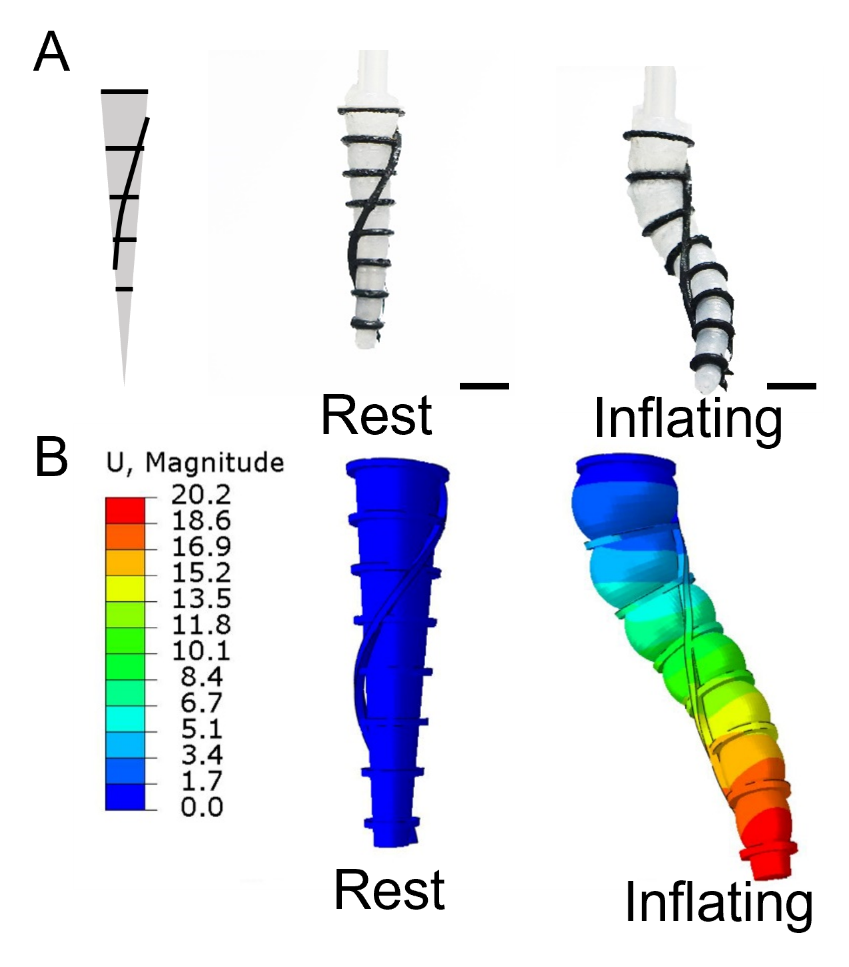


**Fig. S19 Helical bending actuators mimicking the motion of cephalopod tentacles:** (A) By changing the cylinder to a cone tube, the actuator can generate dexterous movement like cephalopods tentacles (Scale bar: 10 mm). (B) FEA simulation of the actuator with a particular fiber pattern.


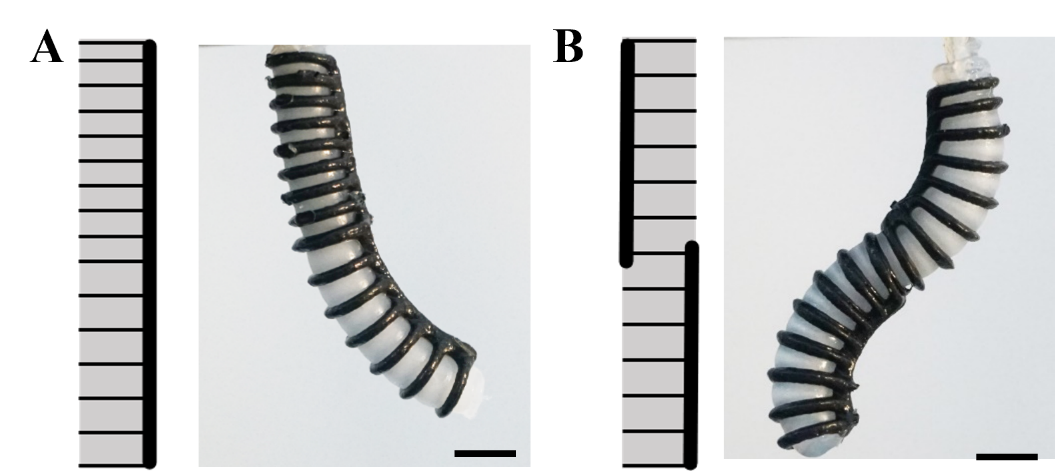


**Fig. S20 Serially connected actuators:** (A) bending curvature of the different parts of the actuator can be realized by adjusting the density of horizontal fibers (Scale bar: 10 mm). (B) The bending direction of the different parts of the actuator can be controlled by the relative positions of constraint fibers (Scale bar: 10 mm).


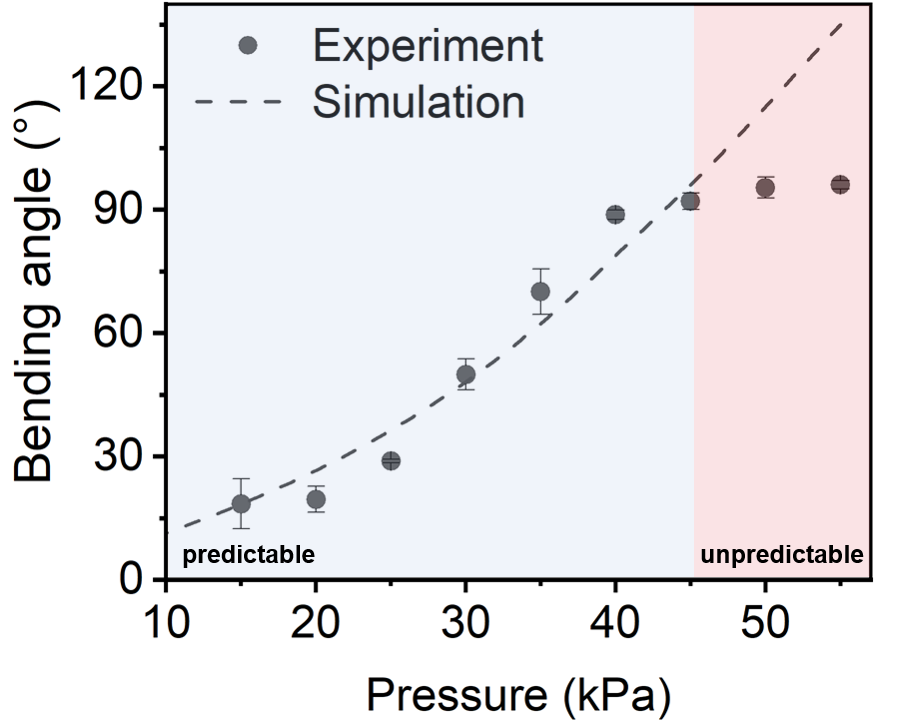


**Figure S21** **Experiment and simulation responses of the bender:** the FEM can’t predict the pneumatic responses well when the strain is too high due to the nonlinear elasticity of material. Therefore, the inverse design pressure is set to be 15 kPa, with which the pneumatic responses of unit actuators are predictable.


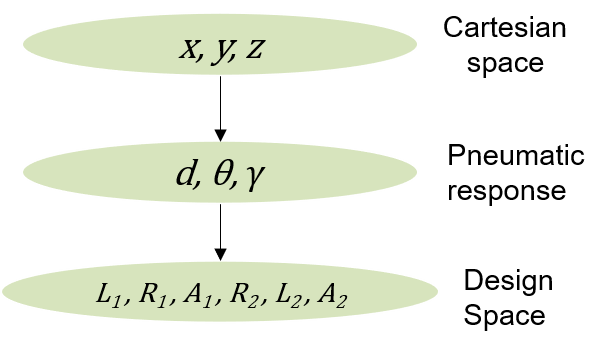


**Fig. S22 The projection flowchart from coordinates in Cartesian space to the design parameters:** In our point position task, the inverse design of serially connected actuators needs two-step of projections. Step 1: Projecting coordinates in Cartesian (*x, y, z*) to the pneumatic response ($d,\theta, and \gamma$). Step 2: Project the pneumatic response ($d,\theta, and \gamma$) to the parameters in the design space (*L_1_, R_1_, L_2_, A_1_, A_2_, R_2_*).


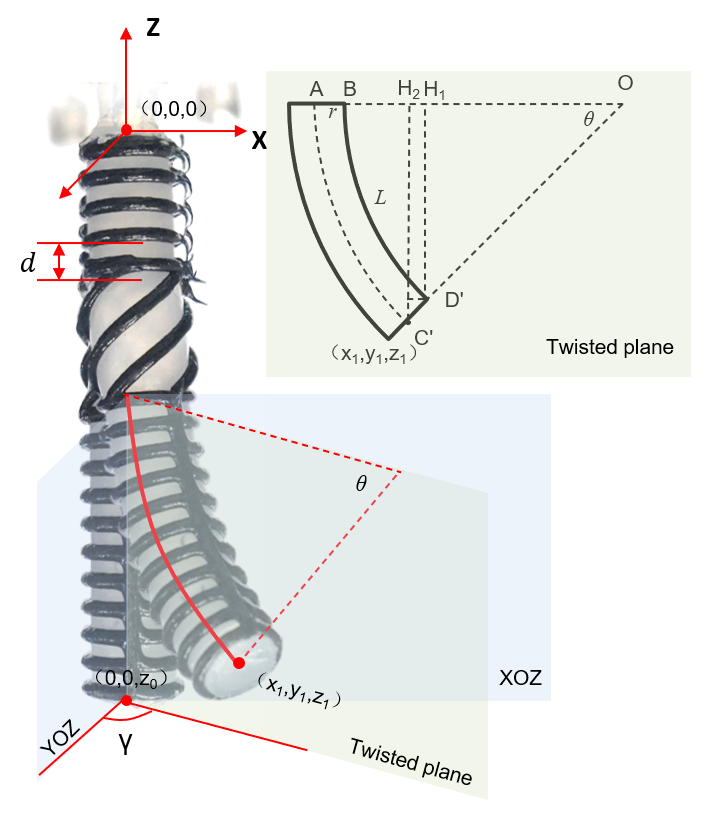


**Fig. S23 Illustration of Inverse kinetic model of actuator designed for point positioning task.**


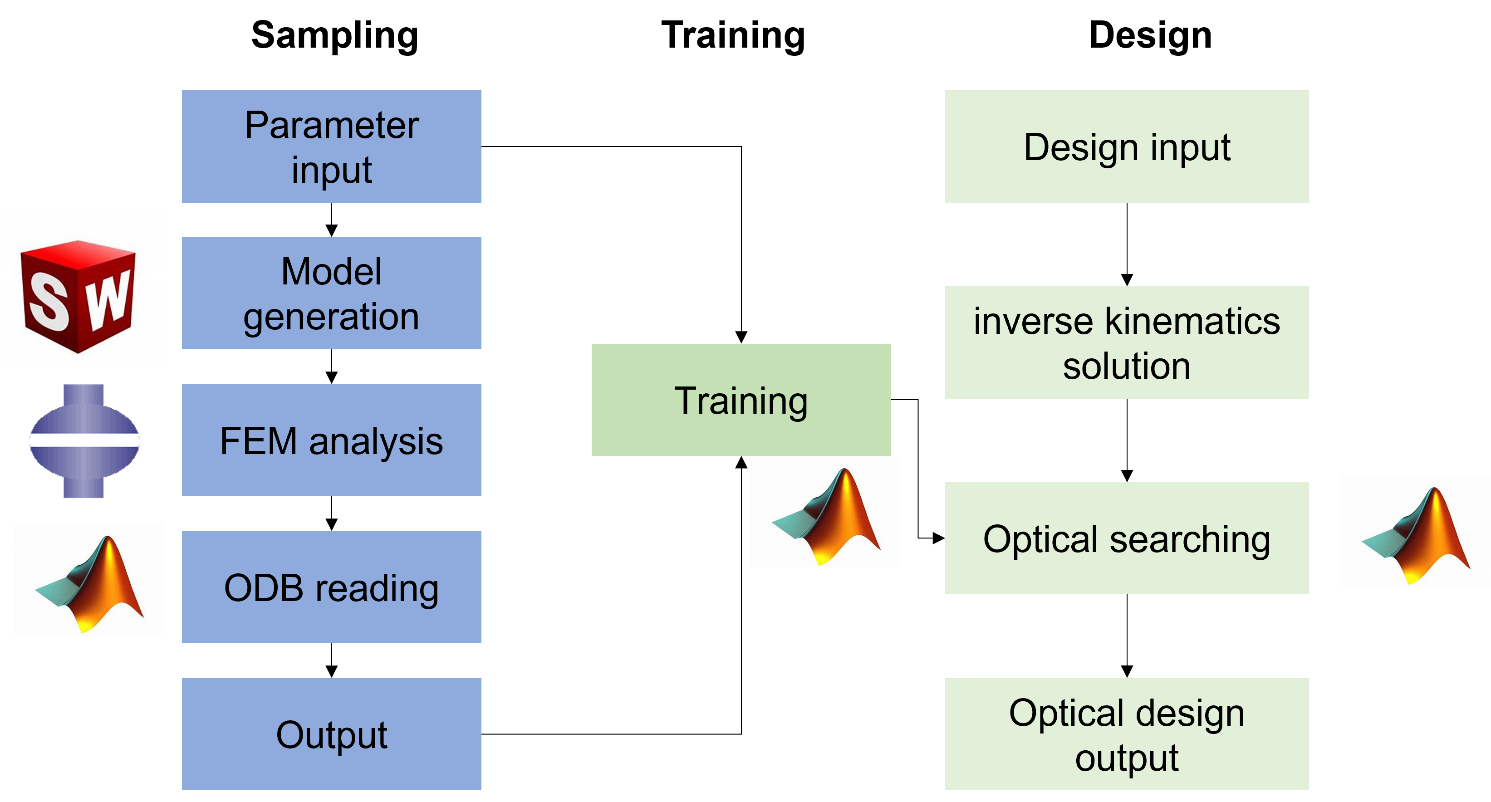


**Fig. S24 Flowchart of our inverse design process:** The inverse design consists of three parts: Sampling, training, and design. (1) Sampling: Three actuators have six design parameters in total (Fig. 4A-C). The ranges for all parameters are ${10mm\leq L}_{1}\leq30mm$, ${0.3\leq R}_{1}\leq0.8$, ${10mm\leq L}_{2}\leq30mm$, ${0.261 rad\leq A}_{1}\leq1.30 rad$, ${0.1\leq R}_{2}\leq0.9$, and ${0.261 rad\leq A}_{2}\leq1.57 rad$. We use isometric sampling in the range (10 sampling points in each parameter). For each actuator, 100 models with different design parameters are then generated in Solidworks software. Then we conduct the FEA of these actuators in the inflating pressure of 15 kPa. A customed MATLAB script is used to read the ODB file, and we get the final response. (2) Training: The surrogate model of the pneumatic response of actuators is established by Kriging’s method using costumed MATLAB scripts. (3) Design: design process consisted of the projection of coordinates in Cartesian space to design pneumatic response and the optimization of design parameters.


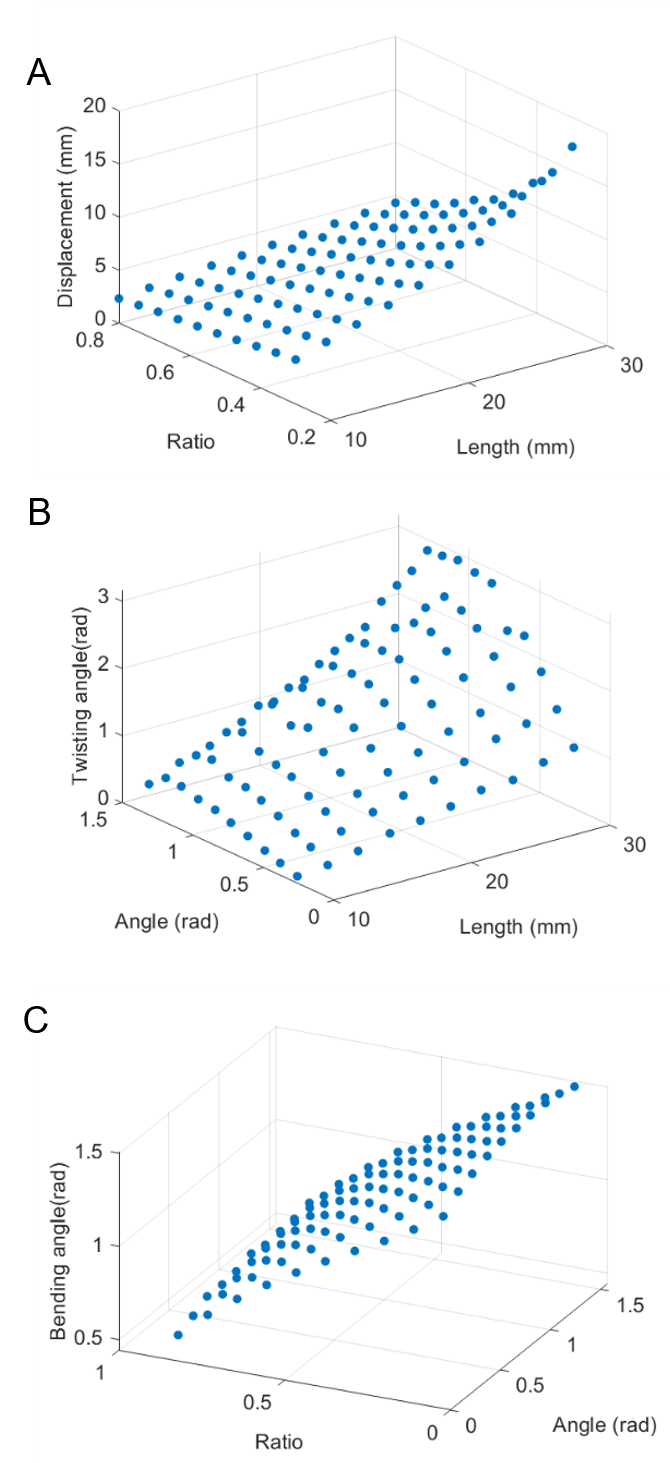


**Fig. S25 Sampling data using the FEA method:** (A) The displacement increases with the increase of the elongator’s length and decrease of the elongator’s ratio. (B) The twisting angle increases as the increase of elongator’s length. However, the twisting angle will increase followed by a decrease with the increase of the leading angle. (C) When the ratio and constrain fiber increase, the bending angle of the bender increases respectively.


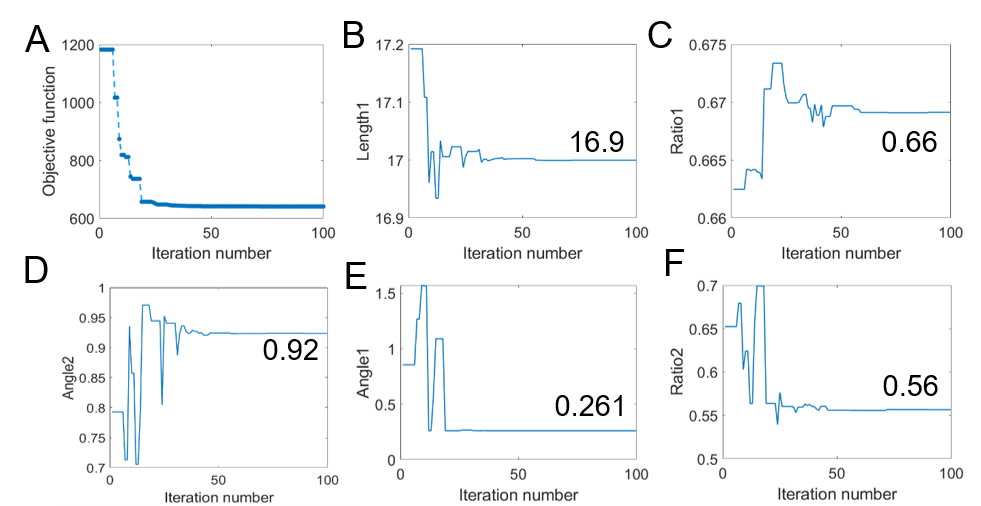


**Fig. S26 The optimization process of the objective function and parameters:** (A) The objective function (volume of hard ink) converge to ~ 650 mm^3^ after ~20 iteration. (B)-(F) The design variable values for L_1_, R_1_, L_2_, A_1_, A_2_, and R_2_ are 16.9 mm, 0.66, 13.1mm, 0.92 rad, 0.261 rad, and 0.56 after ~ 40 iterations.


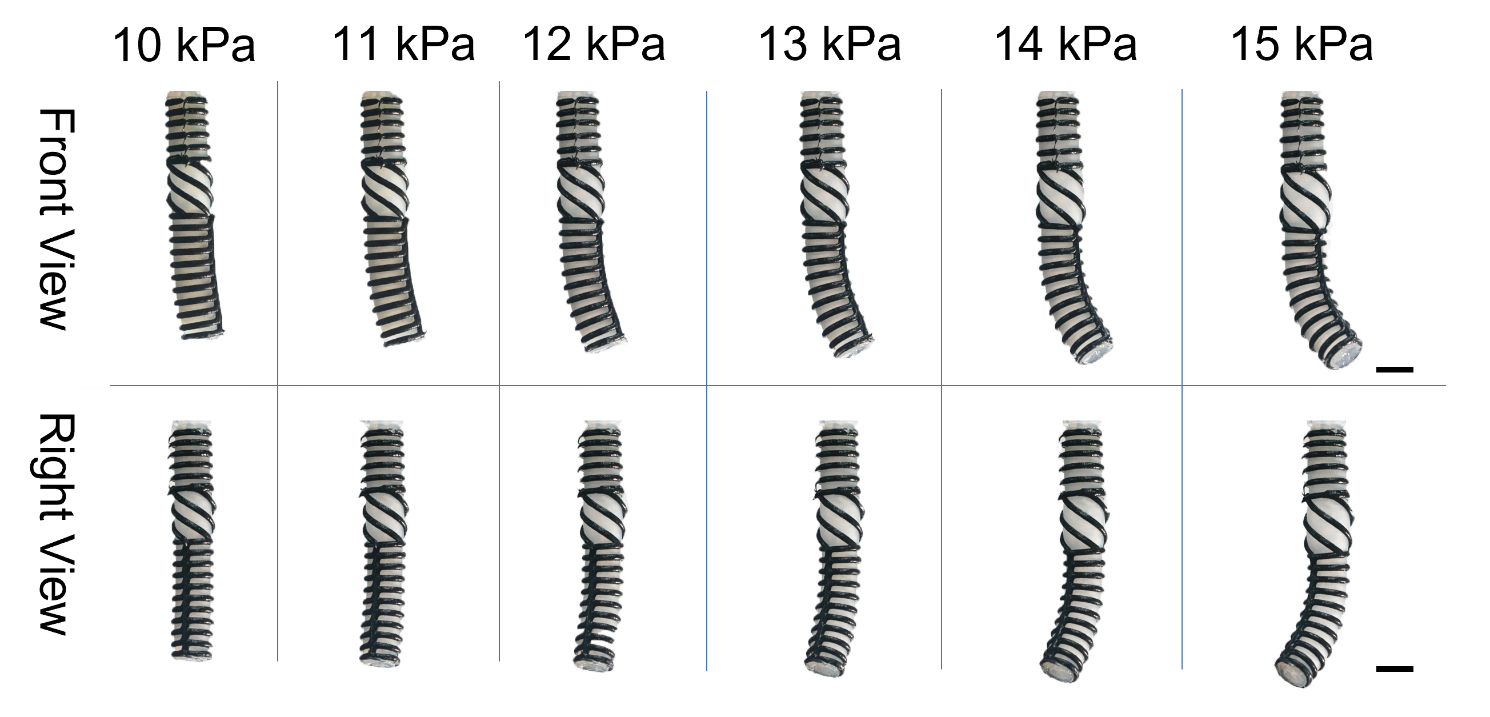


**Fig. S27 Inflating of the serially connected actuator:** The front and right view of the actuator are inflated by 10 – 15 kPa (Scale bar: 10 mm)


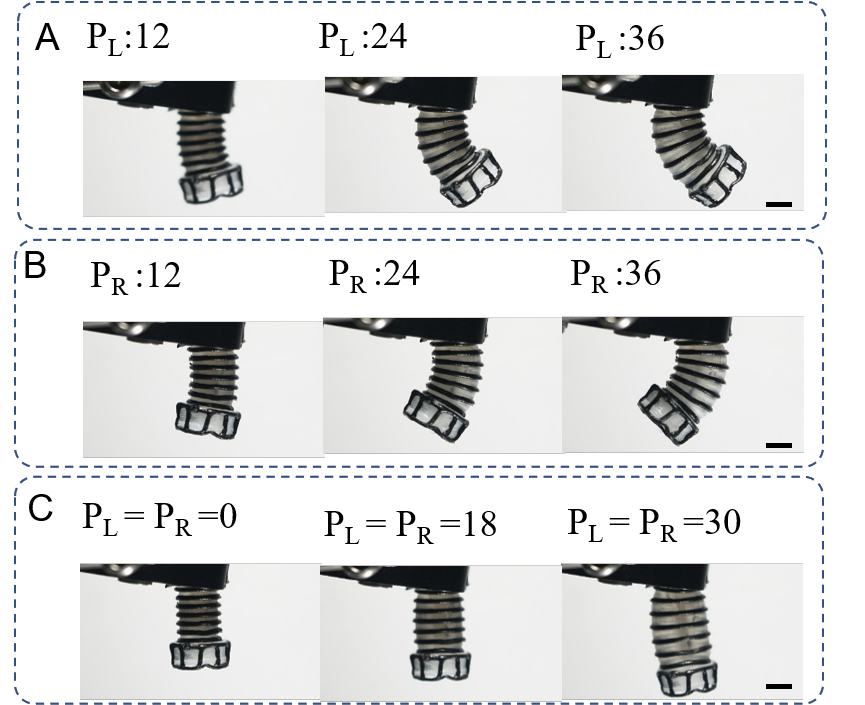


**Fig. S28 Pneumatic response of bidirectional bending actuator under different inflating conditions:** (A) Inflating left channel; (B) Inflating right channel; (C) Inflating both channels (Scale bar: 10 mm).

**Table S1** **Geometry size of actuators and robots**

| Actuator type | | Diameter  (mm) | Length  (mm) | | Thickness of  soft wall  (mm) | | Thickness of fiber layers  (mm) | |  |
| --- | --- | --- | --- | --- | --- | --- | --- | --- | --- |
| Extensor (Fig 4A) | | 14 | | 25 | | 1 | | 1 | |
| Contractor (Fig 4A) | | 14 | | 50 | | 1 | | 1 | |
| Twistor (Fig 4A) | | 14 | | 50 | | 1 | | 1 | |
| Bender (Fig 4A) | | 14 | | 50 | | 1.5 | | 1.5 | |
| Shape morphing actuator (Fig 4D) | | 15 | | 30 | | 1.5 | | 1 | |
| Multi-segment actuator (Fig 5b) | | 11 | | 60 | | 1 | | 0.5 | |
| Climbing robots  (Figure 5A) | Front expander | 21 | | 8 | | 1.5 | | 1.5 | |
|  | Middle extensor | 16 | | 18 | | 1.5 | | 1.5 | |
|  | End expander | 21 | | 8 | | 1.5 | | 1.5 | |

**Table S2** **Comparison between twistors fabricated by different methods**

| Method | | E_hard_  (MPa) | E_soft_  (MPa) | Pressure-bearing  capability (kPa) | Twisting angle*  （°/ mm） | | Torque*  (N∙mm/mm) | |
| --- | --- | --- | --- | --- | --- | --- | --- | --- |
| Tradition casting | Sedal et al. [^S8^] | 1 | 2 | 64** | - | 0.9 | |  |
|  | Connolly et al.^[S9]^ | 1.36 | 31067 | 100** | 0.3 | — | |  |
|  | Connolly et al. ^[S10]^ | 0.48 | 31067 | 60** | 2 | — | |  |
|  | Chen et al. ^[S11]^ | 0.368 | 19.46 | 39** | 0.7 | — | |  |
|  | Yan et al ^[S12]^ *** | 0.12 | ~30000 | 100** | 2 | 0.52 | |  |
| U-based printing | Schaffner et al. ^[S13]^ | 0.13 | 3.4 | 6** | 2.16 | — | |  |
|  | Byrne et al. ^[S14]^ | 0.25 | 120 | 40** | 1 | — | |  |
| ME3P | Our work | 0.15 | 8 | 37 | 3 | 0.6 | |  |

*Normalized by actuators’ length and inflating pressure

**Maximum inflating pressure of the twistor appeared in their work

*** Fiber reinforced actuators function as the units of a twisting actuator

**References**

[S1] Kelessidis, V. C., et al. "Optimal determination of rheological parameters for Herschel–Bulkley drilling fluids and impact on pressure drop, velocity profiles and penetration rates during drilling." Journal of Petroleum Science and Engineering 53.3-4 (2006): 203-224.

[S2] Jiang, Tao, et al. "Extrusion bioprinting of soft materials: An emerging technique for biological model fabrication." Applied Physics Reviews 6.1 (2019): 011310.

[S3] Sacks, Jerome, et al. "Design and analysis of computer experiments." Statistical science 4.4 (1989): 409-423.

[S4] Chen, Hao, et al. "A pointwise ensemble of surrogates with adaptive function and heuristic formulation." Structural and Multidisciplinary Optimization 65.4 (2022): 1-23.

[5] FAC Viana, SURROGATES Toolbox User’s Guide, Version 2.1, http://sites.google.com/site/felipeacviana/surrogatestoolbox, 2010.

[S6] Faramarzi, Afshin, et al. "Equilibrium optimizer: A novel optimization algorithm." Knowledge-Based Systems 191 (2020): 105190.

[S7] Chen, Hao, Weikun Li, and Weicheng Cui. "Disruption-based multiobjective equilibrium optimization algorithm." Computational Intelligence and Neuroscience 2020 (2020).

[S8] Sedal, Audrey, et al. "A continuum model for fiber-reinforced soft robot actuators." Journal of Mechanisms and Robotics 10.2 (2018): 024501.

[S9] Connolly, Fionnuala, Conor J. Walsh, and Katia Bertoldi. "Automatic design of fiber-reinforced soft actuators for trajectory matching." Proceedings of the National Academy of Sciences 114.1 (2017): 51-56.

[S10] Connolly, Fionnuala, et al. "Mechanical programming of soft actuators by varying fiber angle." Soft Robotics 2.1 (2015): 26-32.

[S11] Chen, Shitong, et al. "Topology optimization of skeleton-reinforced soft pneumatic actuators for desired motions." IEEE/ASME Transactions on Mechatronics 26.4 (2021): 1745-1753.

[S12] Yan, Jihong, et al. "A new spiral-type inflatable pure torsional soft actuator." Soft robotics 5.5 (2018): 527-540.

[S13] Schaffner, Manuel, et al. "3D printing of robotic soft actuators with programmable bioinspired architectures." Nature communications 9.1 (2018): 1-9.

[S14] Byrne, Oisín, et al. "Additive manufacture of composite soft pneumatic actuators." Soft robotics 5.6 (2018): 726-736.
